# Genetic determinants of aerial root morphology in Sierra Mixe-derived maize

**DOI:** 10.1007/s00122-026-05348-w

**Published:** 2026-09-05

**Authors:** Daniel Laspisa, Rubens Diogo, Rafael E. Venado, Tess Kern, Natalia de Leon, Jean-Michel Ané, Jason G. Wallace

**Affiliations:** 1 Institute of Plant Breeding Genetics and Genomics, University of Georgia, Athens, GA USA; 2 Department of Bacteriology, University of Wisconsin-Madison, Madison, WI USA; 3 Department of Plant and Agroecosystem Sciences, University of Wisconsin-Madison, Madison, WI USA; 4 Department of Crop & Soil Sciences, University of Georgia, Athens, GA USA; 5 Driscoll’s of Florida, Dover, FL USA; 6 International Maize and Wheat Improvement Center (CIMMYT), El Batán, Texcoco, State of Mexico Mexico

## Abstract

**Key Message:**

We mapped key regions of the maize genome that influence the formation of aboveground (aerial) roots, which in some varieties have been associated with symbiosis with nitrogen-fixing bacteria.

**Abstract:**

Modern agriculture relies heavily on chemically synthesized nitrogen fertilizers, which ensure high yields but also carry high economic and environmental costs. Biological nitrogen fixation (BNF) supplies high amounts of nitrogen to legumes, and several avenues of research are underway to extend it to cereal crops. In maize, aerial roots formed in Sierra Mixe landraces have been associated with BNF. However, much of the genetics underlying aerial root morphology remains unknown. Here, we evaluate aerial root morphology traits associated with BNF in three segregating populations derived from crosses between two Midwest-adapted inbred lines and three landraces. Inclusive composite interval mapping (iCIM) with flowering time as a covariate identified 37 quantitative trait loci (QTL) for three aerial root traits (nodes with roots, root size, and roots per node) which exhibit moderately high heritability (H^2^ = 0.65 to 0.83). The combined proportion of phenotypic variance explained by the detected QTL ranged from 23 to 51%, depending on the trait and population, and potential candidate genes were identified through literature searches, macrosynteny, and gene expression analyses. Introgressing the most relevant aerial root-associated QTL into elite genotypes may provide a path toward achieving meaningful levels of BNF-associated traits in maize, but further work is needed to assess this approach's viability under field conditions.

**Supplementary Information:**

The online version contains supplementary material available at https://doi.org/10.1007/s00122-026-05348-w.

## Introduction

Modern agriculture is heavily dependent on synthetic nitrogen fertilizer produced via the Haber–Bosch process. Although such fertilizers enable high and consistent yields, their use is also associated with many drawbacks, including high costs (Vos et al. 2025; USDA 2024; Erenstein et al. 2022), extensive infrastructure requirements (Snapp et al. 2023), and significant environmental impacts (Goodkind et al. 2023), including water pollution (Liu et al. 2024), eutrophication (Camargo and Alonso 2006), and greenhouse gas emissions (Pan et al. 2022). These challenges underscore the urgent need to reduce dependency on synthetic nitrogen inputs.

One promising avenue lies in harnessing biological nitrogen fixation (BNF) to enhance the growth of cereal crops. BNF, the microbial conversion of atmospheric N_2_ to plant-available NH_4_^+^, has long been recognized as a cornerstone of sustainable nitrogen cycling in natural and agricultural ecosystems (Dixon and Kahn 2004). Unlike legumes, most cereals do not form strong associations with BNF-capable bacteria, which limits their nitrogen-use efficiency (Guo et al. 2023). Recent studies have highlighted the potential of certain traditional maize varieties from southern Mexico to host nitrogen-fixing bacteria on their aerial roots. These "Sierra Mixe" landraces produce a carbohydrate-rich mucilage on these roots, which fosters nitrogen-fixing bacterial communities capable of supplying between 30–80% of the plant's nitrogen needs (Van Deynze et al. 2018; Bennett et al. 2020; Pankievicz et al. 2022). This discovery represented a paradigm shift in cereal BNF research, showing that a structural root modification, rather than a root-zone chemical gradient or endophytic colonization alone, could serve as the primary interface for nitrogen fixation in a grass species under field conditions.

Importantly, aerial root-associated nitrogen fixation is not unique to maize: a parallel system has been described in sorghum (Venado et al. 2025b; Wolf et al. 2024) and an evolutionarily distinct one in mangroves (Inoue et al. 2024). Grass aerial roots remain a unique, yet poorly understood set of lateral organs (Sparks 2023) found only in the tribes Andropogoneae (maize, sorghum, and sugarcane) and Paniceae (foxtail millet) within Poaceae (Hostetler et al. 2021). Some key aerial root traits positively correlate with improved BNF, including the number of nodes forming aerial roots (Van Deynze et al. 2018) and a high number and diameter of aerial roots (Pankievicz et al. 2022). Quantitative trait loci (QTL) and genome-wide association studies (GWAS) have been conducted for brace root-related traits (Sun et al. 2022; Liu et al. 2022; Liu et al. 2023; Singh et al. 2022; Lin et al. 2024; Guo et al. 2021), meaning for aboveground roots that contact and penetrate the ground, but there is a scarcity of research on aerial root traits. Understanding the genetic mechanisms underlying these traits is essential for leveraging them in breeding programs, which, in conjunction with environmental and microbial factors, could enhance nitrogen fixation in maize and other cereals, thereby reducing reliance on synthetic fertilizers and benefiting both the environment and agricultural sustainability (Wen et al. 2021a, b).

Here, we evaluate three populations of recombinant inbred lines (RILs) derived from crosses between two Midwest-adapted inbred lines (PHZ51 or B73) and three landraces from Southern Mexico. We focus on specific aerial root morphology traits that are correlated with mucilage production and thus BNF potential. For example, young aerial roots produce the most mucilage while older ones produce little or none (Pankievicz et al. 2022), so producing more nodes with aerial roots expands not just the total habitat for N-fixing microbes but also the timeframe in which fixation occurs. Larger roots have larger root caps and thus more potential (and actual) mucilage production (Pankievicz et al. 2022). More roots per node could also increase overall habitat for fixation, though it could also result in a net loss of fixation potential if it results in smaller roots. The presence of numerous nodes of large aerial roots was even recognized by the original indigenous communities as being important for these varieties of maize (Van Deynze et al. 2018).

This study estimates the heritability of aerial root traits, examines correlations among these traits, and identifies genomic regions associated with them. This work positions maize aerial root architecture within the broader context of plant–microbe interaction-associated nitrogen fixation systems across grass species, and lays the foundation for pre-breeding efforts to improve BNF-associated traits in maize, paving the way for more sustainable agricultural practices.

## Materials and methods

### Development of mapping populations

Initially, eight crosses were made between an inbred line adapted to the Midwest region of the USA (PHZ51) and eight plants from one of three Oaxacan landraces exhibiting the aerial root-related traits that we were interested in studying (Oaxaca 184 [PI 629238], Oaxaca 182 [PI 645940], and BENZ 638 [AMES 19897; accession numbers are from the United States Department of Agriculture National Plant Germplasm System]). F_1_ individuals were then backcrossed to PHZ51, and BC_1_ plants were subjected to double-haploid (DH) induction in collaboration with Limagrain, resulting in eight preliminary double-haploid populations (DH-I to DH-VIII). Due to logistical constraints, only the two DH populations with the highest number of entries were phenotyped. For notational purposes, we refer to these two DH populations as "Pop 1" and "Pop 2".

Meanwhile, another initial cross was made between a Midwest-adapted line holding an expired Plant Variety Protection Act certificate (B73) and a Chiapaneca landrace (Olotón CHIS684 [CIMMYTMA-10438]; accession number is from the Germplasm Bank at the International Maize and Wheat Improvement Center). F_1_ plants were backcrossed to B73, and then BC_1_ plants were subjected to five generations of self-pollination using the single-seed descent (SSD) method (no selection applied) to obtain 87 recombinant inbred lines with high levels of homozygosity. For notational purposes in this article, we named these BC_1_-SSD_5_ plants as "Pop 3".

### Genotyping the mapping populations

DH plants from Pops 1 and 2 (236 and 248 lines, respectively) were genotyped in collaboration with Limagrain using a chip with 1,800 single-nucleotide polymorphism (SNP) markers. Genotypes were imputed using the Tassel5 FSFHap command-line plugin (Swarts et al. 2014). They were then filtered to remove non-mapped or non-segregating sites. Markers without correspondence in the B73 v5 reference genome were excluded, as were non-polymorphic markers, those with missing data greater than 20% and/or a minimum allele frequency lower than 10%. Based on genotypic information for the common parent (PHZ51) obtained using the same chip, the genotyping data for Pops 1 and 2 were then converted from the nucleotide-based HapMap format to a 0/1/2 coding format.

DNA samples from the 87 BC_1_S_5_ lines from Pop 3 were sent to SAGA (Servicio de Análisis Genético para la Agricultura) at CIMMYT for SNP detection using Diversity Array Technology sequencing (DArTseq) (Edet et al. 2018). This method uses a two-enzyme digestion to generate reduced-representation libraries, followed by sequencing of the resulting fragments. Approximately 110,000 SNP-containing sequences (reference and alternative alleles) were converted to FASTA format and aligned to the B73 v4 reference genome. Only SNPs where both alleles aligned uniquely to the same genomic location were retained. The data were then converted to HapMap format and analyzed using TASSEL5 (Bradbury et al. 2007). SNPs with a minor allele frequency < 0.1 or missing data in > 60 individuals were filtered out, resulting in around 5,400 polymorphic SNPs. Genotypes were imputed and converted to a 0/1/2 coding format using FSFHap. Later, SNP positions were lifted over (from B73 v4 to v5 reference genomes) using the *rtracklayer* package (Lawrence et al. 2009) in RStudio (R Core Team 2024) using a chain file downloaded from the Maize Genome Database (MaizeGDB): https://download.maizegdb.org/Zm-B73-REFERENCE-NAM-5.0/chain_files/.

### Genetic map construction

The genetic maps of the three populations were constructed using the MAP function of the QTL IciMapping software (Meng et al. 2015). Genetic maps were validated by comparing the positions of the genetic map to the B73 v5 physical map.

### Field experiments and phenotyping

Pops 1 and 2 were grown in Watkinsville, Georgia (33°43′ 25.9″ N, 83°18′ 19.8″ W) and Verona, Wisconsin (43°03′ 56.0″ N, 89°32′ 34.0″ W) in 2023 and 2024 in a randomized complete block design with two replicates and either 12 (Georgia) or 20 (Wisconsin) seeds sown per plot. Pop 3 was grown only in Verona in 2023 and 2024. Plots measured 3.0 m (Georgia) or 3.8 m (Wisconsin) long by 0.75 m wide with 1.8 m alleys separating ranges of plots. Three to four plants within the plots (i.e., not subject to any edge effect) were assessed, and the following aerial root traits were measured after anthesis but before senescence: number of nodes forming aerial roots, number of aerial roots per node, average diameter of those aerial roots, stalk diameter, days to anthesis, and flag leaf height.

The number of nodes forming aerial roots was measured on individual plants on the central stalk (no tillers). Beginning at the soil level, we counted the number of nodes with visible aerial roots, including those with roots eventually touching the soil. Nodes were counted if the aerial roots extended at least 1 cm from the stalk. A rating of 0.5 was assigned when bumps (< 1 cm in length) were developing on the node right above. For example, if a plant has four nodes with aerial roots longer than 1 cm and node five has root bumps, the number of nodes was recorded as 4.5. The number of aerial roots per node was measured on individual plants, counting on node four. If the plant had fewer than four nodes with aerial roots, the number of roots on the highest complete node was counted. A “complete node” was defined as one in which there were aerial roots longer than 1 cm surrounding the entire node. If the top-most node with aerial roots was incomplete, we counted the number of roots in the node right below (provided it was complete). The aerial root diameter was measured on three roots per plant using digital calipers on three non-adjacent aerial roots from node four (or the highest node if the plant did not have four nodes with aerial roots), approximately 1–2 cm from the stalk. The stalk diameter was measured on individual plants using the same caliper across the narrower width of the elliptical stalk between nodes three and four. The number of days from planting to anthesis was recorded as the date when 50% of a plot exhibited anther exertion (with consequent pollen release) on more than half of the central tassel spike. The flag leaf height was measured on individual plants and refers to the attachment point of the flag leaf to the maize stalk.

Data quality was assessed by inspecting distributions for each trait within each environment, manually correcting typos and removing records from plants that showed evidence of mechanical damage, lodging, high infestation of corn smut (caused by the fungus *Ustilago maydis*), and from plots affected by flooding (field spots with low drainage) or low establishment (with <6 plants). The following allowable phenotype ranges were established for each trait and values outside these ranges were excluded as likely notation errors: number of nodes forming aerial roots (0–9), aerial root diameter (1–10 mm), number of aerial roots per node (3–40), stalk diameter (10–40 mm), days to anthesis (60–120), and flag leaf height (50–350 cm)‬.

### Heritability estimation

Phenotypic-based broad-sense heritability (H^2^) was obtained via linear mixed models using the *lme4* package (Bates et al. 2015). Because field trials followed a completely randomized design (CRD), the random-effects structure for plant/aerial-root-level models included genotype, environment, genotype × environment interaction (G × E), and plot nested within G × E. Variance components were estimated using restricted maximum likelihood (REML). To prevent the conflation of physically distinct plots across environments, plot IDs were re-coded as unique location-year-plot composites prior to model fitting. H^2^ on an entry-mean basis was calculated to adjust for unbalanced replication as:

$$H^{2} = \frac{{\sigma_{G}^{2} }}{{\sigma_{G}^{2} + \frac{{\sigma_{GE}^{2} }}{{\overline{e}}} + \frac{{\sigma_{Plot}^{2} }}{{\overline{e} \cdot \overline{r}}} + \frac{{\sigma_{\varepsilon }^{2} }}{{\overline{e} \cdot \overline{r} \cdot \overline{m} }}}}$$

where ē, r̄, and m̄ represent the harmonic means of environments per genotype, plots per G × E combination, and measurements per plot, respectively (Holland et al. 2003; Piepho & Möhring 2007). For days to anthesis, which was scored once per plot, the plot-level random effect was omitted because plot-to-plot variation constitutes the residual error. Its H^2^ was calculated as:

$$H^{2} = \frac{{\sigma_{G}^{2} }}{{\sigma_{G}^{2} + \frac{{\sigma_{GE}^{2} }}{{\overline{e} }} + \frac{{\sigma_{\varepsilon }^{2} }}{{\overline{e} \cdot \overline{r}}}}}$$

For the estimation of SNP panel-based genomic heritability (h^2^_SNP_), best linear unbiased estimates (BLUEs) of genotype means were initially obtained via linear mixed models using the *lme4* package (Bates et al. 2015). Genotype was fitted as a fixed effect (within the same random background: environment, G × E, and plot, where applicable) to avoid a second round of shrinkage during subsequent genomic modeling (Holland and Piepho 2024). Genotype BLUEs were post-hock analyzed using the *emmeans* package (Lenth 2024). By fitting models directly on plant/root-level records instead of pre-averaged plot means, plots with fewer measurements contributed proportionally less to the plot random effect without requiring explicit weighting. An additive genomic relationship matrix was constructed from SNP markers via the A.mat() function in the *rrBLUP* package (Endelman 2011). Genotype effects were then modeled as random with covariances proportional to this matrix using the mixed.solve() function. Finally, h^2^_SNP_ was calculated as:

$$h_{SNP}^{2} = \frac{{\sigma_{A}^{2} }}{{\sigma_{A}^{2} + \sigma_{\varepsilon }^{2} }}$$

where σ^2^_A_ is the marker-based additive genetic variance and σ^2^_ε_ is the residual variance from the genomic model. Both heritability metrics (H^2^ and h^2^_SNP_) are reported to provide a complete overview of trait genetic architecture and mapping power.

### Pairwise correlation

Pairwise Pearson correlations were calculated among the six aerial root and agronomic traits using plant/root-level raw phenotypic measurements. To reduce pseudoreplication due to unequal within-plant subsampling, raw records were first averaged across aerial root replicates within each plant, then across plants within each plot, and finally averaged across plots to yield one mean per genotype-environment combination. Pearson correlation coefficients were computed for each pairwise combination of traits using pairwise-complete observations, such that missing data for one trait did not exclude available observations for other trait pairs. Significance was assessed via two-tailed tests (*p* < 0.05), with populations and environments pooled across the full dataset.

### Phenotypic adjustment for flowering time

To remove the confounding effect of flowering time on aerial root morphology traits prior to QTL mapping, nodes with aerial roots, aerial root diameter, and aerial roots per node were each residualized against days to anthesis (DTA) in a single-stage linear model fit to the plot-level means. This procedure was done separately for each population:

Trait∼Environment+DTA+Genotype

where Environment and DTA were fitted as covariates and Genotype was fitted as a fixed effect. Trait and DTA values were computed as weighted plot-level means, obtained by averaging raw plant-level (and, for aerial root diameter, root-level) measurements within each plot. Genotype-level estimated marginal means (EMMs), adjusted for environment and DTA, were extracted using the *emmeans* package (Lenth 2024) and used as input for downstream QTL mapping.

The effectiveness of the residualization procedure was evaluated by comparing the distributions of raw data and residuals after DTA removal through histograms and quantile–quantile (Q–Q) plots, and by confirming homoscedasticity and the absence of systematic trends through inspection of residual plots for each trait.

### QTL mapping

We mapped QTL for aerial root traits using the BIP function (developed for biparental populations) in IciMapping software, version 4.2 (Meng et al. 2015). Parameters were adjusted as follows: mapping method = ICIM-ADD (based on additive effects); step size = 1 cM; probability of inclusion (PIN) = 0.001; LOD threshold based on 1,000 permutations; α (type I error) = 5%. This model was used to extract the logarithm of the odds (LOD) scores, the left and right markers that limit each identified QTL, their confidence intervals, the percentage of phenotypic variation explained by each QTL (PVE%), and their additive effects.

To provide a more accurate estimate of the total PVE% for each trait and population, a joint model was fitted in which all confirmed QTL were included as simultaneous predictors. For each trait × population combination, the marker physically closest to each QTL’s peak was used as a proxy for that QTL, and a linear model was fitted as:

$$Adjusted \, mean\sim Mar\ker \_QTL_{1} + Mar\ker \_QTL_{2} + \, ... \, + Mar\ker \_QTL_{n}$$

The joint model adjusted R^2^, which penalizes for the number of predictors, was reported as the total phenotypic variance explained (PVE).

### Macrosynteny analysis

The maize gene file Zm-B73-REFERENCE-NAM-5.0_Zm00001eb.1.gff3.gz file was downloaded from MaizeGDB (https://download.maizegdb.org/Zm-B73-REFERENCE-NAM-5.0/) (Woodhouse et al. 2021a). The reference genomes files for two related grasses, sorghum (*Sorghum bicolor*; Sbicolor_730_v5.1.gene.gff3) and foxtail millet (*Setaria italica*; Sitalica_312_v2.2.gene.gff3) were downloaded from the Plant Comparative Genomics portal of the Department of Energy’s Joint Genome Institute (https://phytozome-next.jgi.doe.gov/) (Goodstein et al. 2012). Orthologous gene lists were compiled from curated orthology data hosted in the BioMart tool at Ensembl Plants database (https://plants.ensembl.org/index.html). QTL-based syntenic relationships (macrosynteny) were evaluated using a collinearity analysis anchored on the QTL previously identified for aerial root traits across the three mapping populations. For each QTL confidence interval, all annotated maize genes located within the interval were retrieved and ordered by physical position, and their orthologs in sorghum and foxtail millet were identified and ordered by physical position on the corresponding chromosome of the target species. Because maize underwent a whole-genome duplication, a subset of maize genes had more than one ortholog in each target species; all ortholog copies were retained as independent data points rather than restricting the analysis to a single best match. Orthologous loci that could not be unambiguously assigned to a chromosome in the target genome assembly (e.g., loci placed on unplaced scaffolds) were excluded from the analysis. For each QTL × species combination, the target-species chromosome harboring the largest proportion of the retrieved orthologs (the “majority chromosome”) was identified, and the Spearman rank correlation coefficient (ρ) between maize gene order and ortholog physical position was calculated, restricted to orthologs on that chromosome. A genomic region was classified as syntenic ("collinear") when at least 60% of its retrieved orthologs mapped to a single majority chromosome and the absolute value of ρ was ≥ 0.7; a negative ρ indicates a collinear block in inverted orientation relative to maize. Macrosynteny relationships were visualized using the *ggplot2* package (Wickham 2016) as a genome-wide ideogram depicting the ten maize chromosomes and, for each target species, the collinearity strength (|ρ|, represented by tick-mark opacity) and formal syntenic classification (collinear vs. not collinear) relative to maize.

### Comparison to genes or loci previously associated with nodal root traits

QTL observed in our populations were compared with 280 QTL/GWAS hits from twelve publications (Burton et al. 2014a; Burton et al. 2014b; Moussa et al. 2021; Sun et al. 2022; Sun et al. 2020; Ku et al. 2012; Wang et al. 2019; Gu et al. 2017; Cai et al. 2012; Zhang et al. 2018; Liu et al. 2023; Chen et al. 2023) reporting traits related to maize nodal (shoot-born) roots, that is, crown or brace roots. Coordinates of published QTL/GWAS hits were converted to B73 v5 physical map coordinates (Hufford et al. 2021) to validate overlap.

A list of genes potentially involved in the formation and growth of nodal roots was also compiled from results in maize (Chen et al. 2021; Cowling et al. 2023; Hochholdinger et al. 2018; Hostetler et al. 2021; Li et al. 2019; Liu et al. 2022; Suzuki et al. 2015; Xu et al. 2015; Yang et al. 2022; Zhu et al. 2022; Zhang et al. 2015; Huang et al. 2017) and sorghum (Wolf et al. 2024). Gene coordinates from older genome assemblies were converted to the B73 v5 reference genome using the Translate Gene Model IDs tool from Maize Genome Database (https://www.maizegdb.org/gene_center/gene). Sorghum gene sequences were downloaded from SorghumBase (https://www.sorghumbase.org/) and used as queries in the BLAST tool of MaizeGDB (https://www.maizegdb.org/BLAST). Annotated maize genes with more than 80% similarity to query regions were considered potential orthologs of the respective sorghum genes.

### In silico gene expression analyses

Using the Gene Data tool from MaizeGDB (https://www.maizegdb.org/gene_center/gene), we first downloaded all the gene models whose positions are within the QTL intervals we identified. Next, we downloaded gene expression data from various tissues (primarily nodal roots and internodes) for those genes derived from publicly available maize developmental atlases (Stelpflug et al. 2016; Walley et al. 2016; Sekhon et al. 2012) on the qTeller platform (https://qteller.maizegdb.org/) (Woodhouse et al. 2021b). Their pipeline mapped RNA-seq reads with STAR (Dobin and Gingeras 2015) and quantified expression with Cufflinks (Trapnell et al. 2012), producing Fragments Per Kilobase of transcript per Million (FPKM) values for each gene. We compared gene expression data from aerial roots originated in the first node above ground (Stelpflug et al. 2016 called them *brace roots from node 6*), which were in earlier stages of elongation and thickening, with: (i) Data from the fourth elongated internode during phenological state V9; (ii) Data from crown roots growing along the soil line (Stelpflug et al. 2016 called them *crown roots from node 5*), which were already fully elongated during phenological state V13. We excluded genes with FPKM values < 1 across all three conditions to retain only truly induced and repressed genes, while removing low-expression noise and avoiding FPKM biases. Then, genes whose expression showed an absolute Log_2_ fold change (Log₂ FC) > 2 (that is, a fold change >4 or <0.25) in both comparisons (expression in aerial roots vs young internodes, and in aerial roots vs crown roots) had their gene expression data detailed in a broader range of nodal roots and internode tissues (Stelpflug et al. 2016; Walley et al. 2016) through an Euclidean-clustered heatmap.

## Results

### Genotyping and genetic map construction

Eight BC_1_-derived doubled-haploid (DH) lines (DH-I to DH-VIII) were created after eight initial crosses between PHZ51 and selected plants from three landraces from the Oaxaca region of Mexico. Their relatedness was checked by multidimensional scaling (MDS), and we do not observe any subclusters or individuals that suggest genetic contamination (Supplementary Figs S1–S2). The overall distribution of minor allele frequencies is consistent with the expected BC_1_ parameters (Supplementary Fig S3).

Among the eight populations, two (DH-I and DH-VII), which we refer to hereafter as Pop 1 and Pop 2, were selected to be used as mapping populations (Supplementary Table ST1). When genotyping both populations, 1,372 variable sites were identified; after removing low-quality sites, Pop 1 retained 643 markers, while Pop 2 retained 646 (Supplementary Tables ST2-ST3). Our third mapping population (Pop 3) is a BC_1_-SSD_5_ made between B73 and Olotón CHIS684 (CIMMYTMA-10438). It was genotyped using Diversity Array Technology (DArT) sequencing, resulting in 1,145 informative markers (Supplementary Table ST4). Genetic maps for the three populations were validated by comparing marker positions with Zm-B73-REFERENCE-NAM-5.0 physical map (Supplementary Fig S4).

Although the overall allele frequencies were consistent with BC_1_-derived populations, segregation distortion analysis across the three mapping populations reveals distinct patterns of departure from expected Mendelian ratios at specific locations. 38.3%, 37.6%, and 10.2% of markers deviated significantly from the expected 3:1 ratio at *p* < 0.05, reflecting both the contrasting population structures and the genetic distances between the parental lines involved (Supplementary Fig S5). Nine putative segregation distortion regions (SDRs) across six chromosomes in Pops 1 and 2, plus one SDR in Pop 3, were identified (Supplementary Table ST5). All markers were retained in the linkage maps to avoid creating large gaps potentially detrimental to QTL detection.

### Phenotypic variation, heritability, and trait correlations

**Fig. 1:**
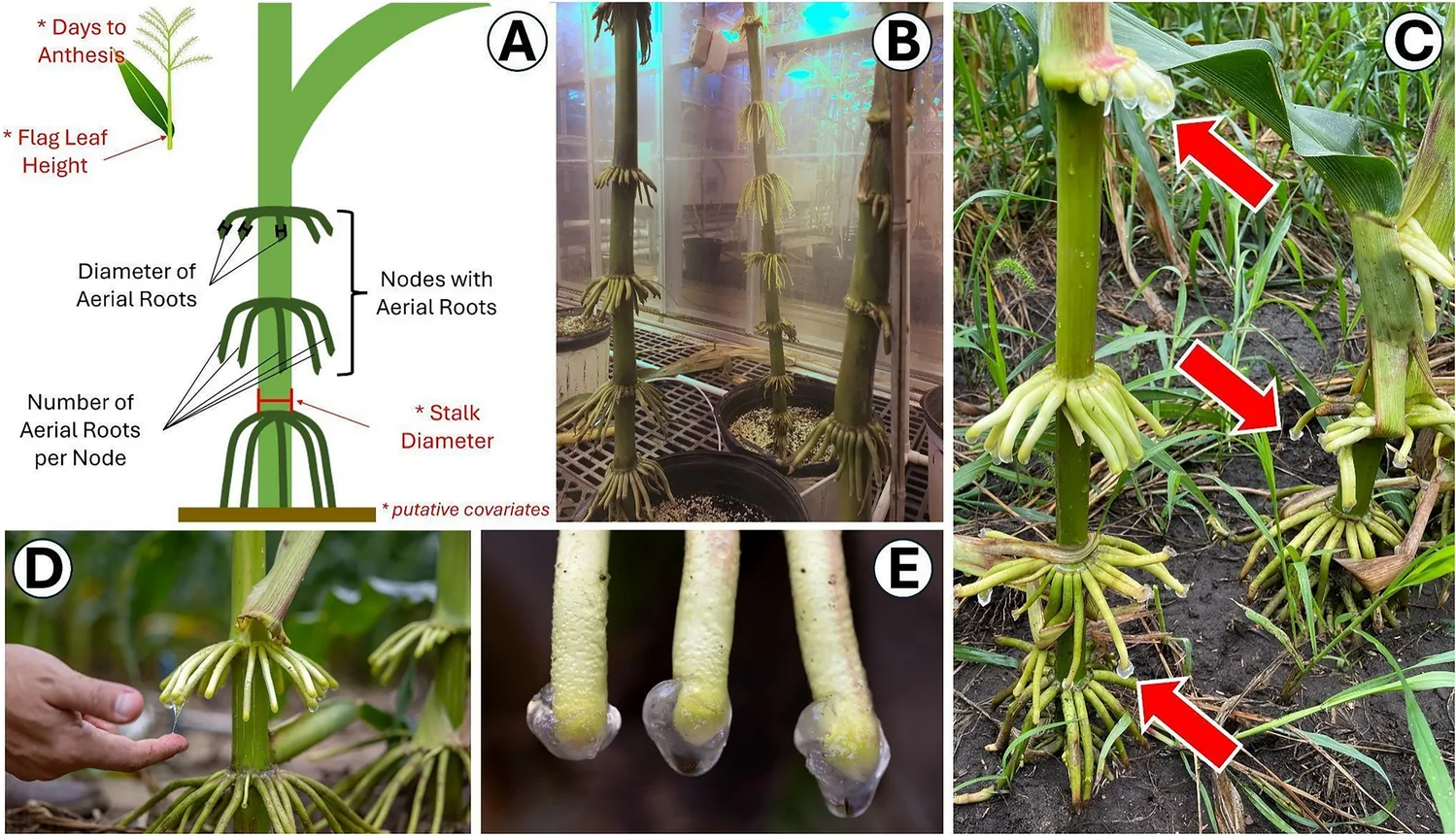
Aerial root phenotypes. **A** Schematic representation of the six traits evaluated in this study. Aerial root traits (number of nodes with roots, roots per node, root diameter) have been correlated with increased mucilage production (Van Deynze et al. 2018; Pankievicz et al. 2022), so they can be considered proxies for nitrogen fixation in the mucilage. Stalk diameter, days to anthesis, and flag leaf height were measured as putative covariates. **B** Oaxa524 plants in the greenhouse showing many nodes with aerial roots. **C**–**E** Different genotypes cultivated under field conditions; the mucilage produced at the tips of the aerial roots is highlighted in all three photos

Pops 1 and 2 were planted in two years (2023 and 2024) in both locations (Wisconsin and Georgia); Pop 3 was only planted in Wisconsin in these years. Phenotyped traits are illustrated in Fig. 1. Data were collected from four randomly selected, non-edge plants (Supplementary Table ST6). Across the three mapping populations (563 lines in total), 537 lines were consistently phenotyped (i.e., two replicates per environment) in 2023 and 519 in 2024 in Wisconsin; in Georgia (where only Pops 1 and 2 were evaluated), this number was 350 in 2023 and 432 in 2024. Missing genotypes were due to insufficient seed, low stand count (< 6 plants per plot), mechanical damage, or lodging.

**Fig. 2:**
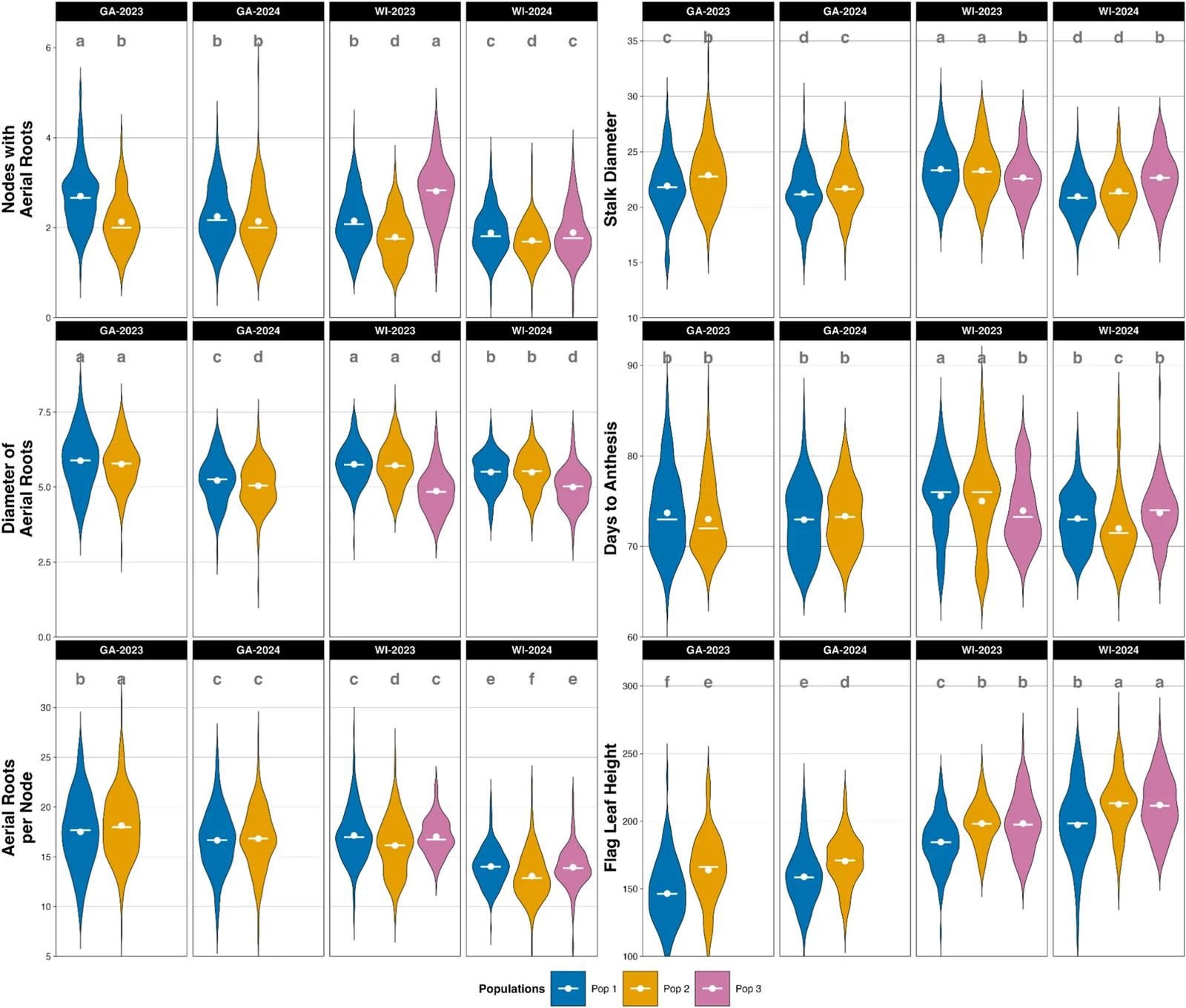
Violin plots of phenotypes separated by trait, year, location, and population. Each group of plots shows the distribution of values for individual traits (labeled at left), separated by environment (boxes), and population (colors). White circles indicate the mean for each group, and a white horizontal line denotes the median. The Scott-Knott clustering algorithm was applied at a 5% significance level to compare means, and lowercase letters above each violin show the grouping of means

Phenotypic ranges for each population are shown in Fig. 2. Plants from Pops 1 and 3 form more nodes with aerial roots than Pop 2. In contrast, plants from Pop 2 are taller than those from Pop 1 and tend to form thicker stalks, which is most evident in Georgia. Plants from Pop 3, in turn, exhibit thinner aerial roots than those from Pops 1 and 2. Although the number of aerial roots per node tends to be statistically similar in Georgia, Pops 1 and 3 exhibit higher values for this trait than Pop 2 in Wisconsin. The number of nodes with aerial roots is lower in Wisconsin than in Georgia in both 2023 and 2024. In contrast, plants in Wisconsin grow much taller and tend to flower later, likely due to photoperiod differences between the locations. Aerial root diameters, as well as the number of aerial roots per node and the stalk diameter, were higher in 2023 compared to 2024 across both locations.

**Fig. 3:**
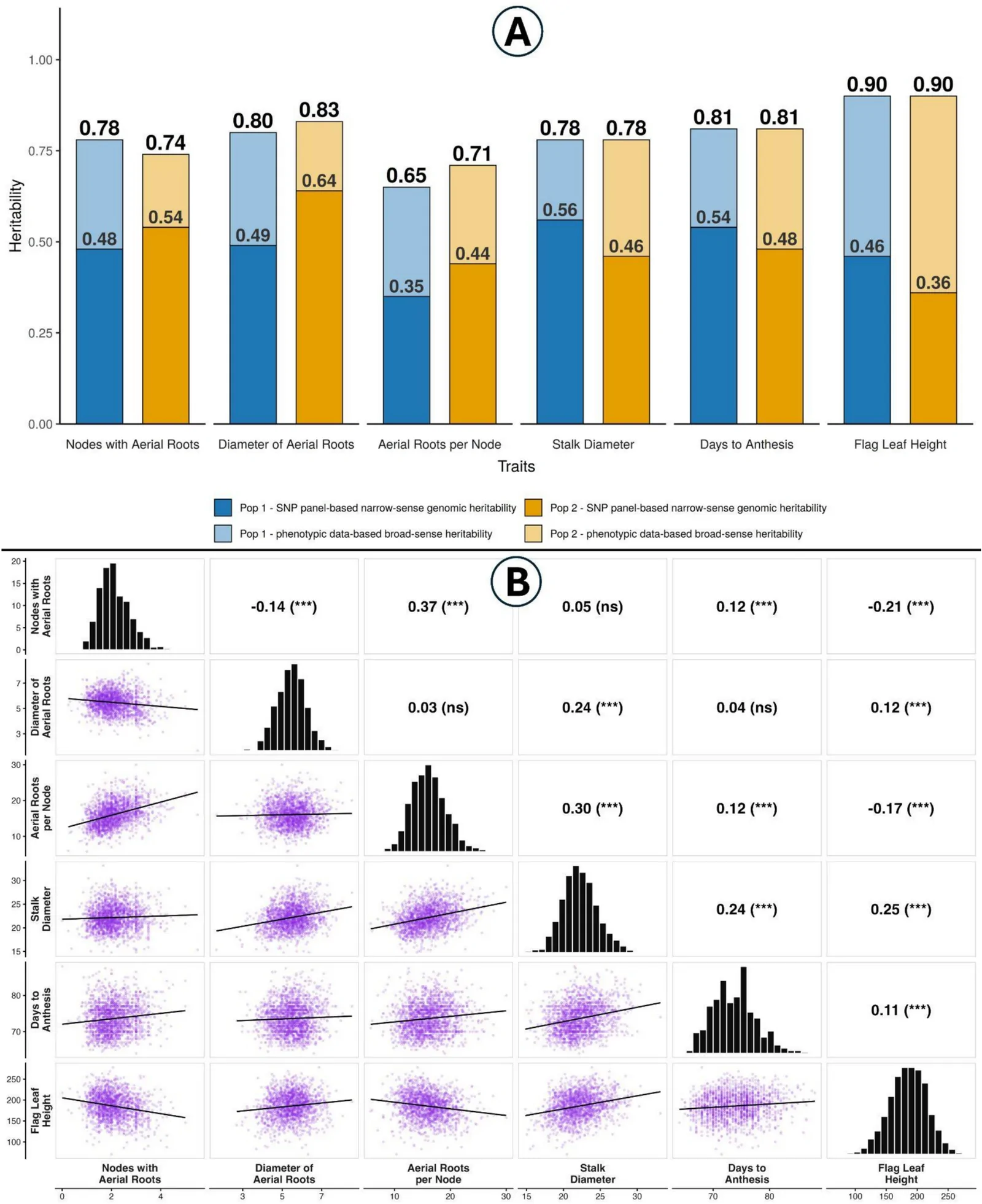
Heritability and pairwise correlation analyses for three aerial root traits and three putative covariates. **A** Phenotypic-based broad-sense heritability (H^2^, illustrated as full bar heights) was estimated on an entry-mean basis after combining results across locations (Wisconsin and Georgia) and years (2023 and 2024) for Pops 1 and 2; Pop 3 was excluded because it was only measured in one location. SNP-based narrow-sense genomic heritability (h^2^_SNP_, darker bars) was calculated as the proportion of variance captured by the genetic markers. **B** The histograms plotted diagonally represent the frequency distribution of phenotypes for each trait, as a percentage of occurrence (y-axis omitted for this data). The plots below the histograms are scatterplots of two traits. The x- and y-axes of the scatterplots represent the observed values for each trait, with a regression line. The values just above the diagonal axis represent Pearson's correlation coefficients between the two traits. In parentheses are the calculated p-values (correlation significance to test linear association): ^ns^ for *p* > 0.05, * for *p* < 0.05, ** for *p* < 0.001, and *** for *p* < 0.001

The phenotypic-based broad-sense heritability (H^2^) values for aerial root traits are moderately high (ranging from 0.65 to 0.83 in Pops 1 and 2), with aerial root diameter being the most heritable (average H^2^ ≈ 0.815) (Fig. 3A). The number of nodes with aerial roots has an average H^2^ of 0.76, while the number of aerial roots per node is the most environmentally dependent, with an estimated H^2^ of 0.68. Average H^2^ values for three putative covariates (stalk diameter, days to anthesis, and flag leaf height) were also estimated: 0.78, 0.81, and 0.9, respectively (Supplementary Table ST7). The SNP-based narrow-sense genomic heritability (h^2^_SNP_) values ranged from 0.35 to 0.64 across the three aerial root traits in Pops 1 and 2. The order remained the same: aerial root diameter showed the highest value (average h^2^_SNP_ = 0.565), followed by the number of nodes with aerial roots (0.51), and the number of aerial roots per node (0.395). Among the putative covariates, stalk diameter and days to anthesis exhibited an average h^2^_SNP_ of 0.51, followed by flag leaf height (0.41) (Supplementary Table ST8).

Pairwise comparison reveals moderate correlations between between the number of nodes with aerial roots and the number of aerial roots per node (Pearson’s r = 0.37), between stalk diameter and the number of aerial roots per node (r = 0.3), and between stalk diameter and aerial root diameter (r = 0.24). Days to anthesis were slightly positively correlated with the number of nodes with aerial roots (r = 0.12) and with the number of aerial roots per node (r = 0.12). The flag leaf height, in turn, is slightly negatively correlated with the number of nodes with aerial roots (r = − 0.21) and the number of aerial roots per node (r = − 0.17). The correlation between the number of aerial roots per node and aerial root diameter is statistically non-significant (Fig. 3B).

Plot-level means for aerial root traits were obtained before and after the residualization against DTA (Supplementary Table ST9). Across all three traits and all population × environment combinations, the Shapiro–Wilk *p*-value increased after DTA covariate removal in 9 out of 10 cases each for nodes with aerial roots and aerial roots per node, and in 3 out of 10 cases for aerial root diameter (Supplementary Figs S6-S8). The standard deviation (SD) values were nearly identical between raw and residual distributions across all combinations, with a mean absolute difference of 0.010, 0.005, and 0.042 for nodes with aerial roots, aerial root diameter, and aerial roots per node, respectively (Supplementary Table ST10), confirming that the DTA covariate removed developmental timing variance without substantially compressing the genetic variance available for QTL detection. Later, genotype-level estimated marginal means (EMMs), adjusted for environment and DTA, were extracted for downstream QTL mapping (Supplementary Table ST11).

### QTL mapping for aerial root traits

**Fig. 4:**
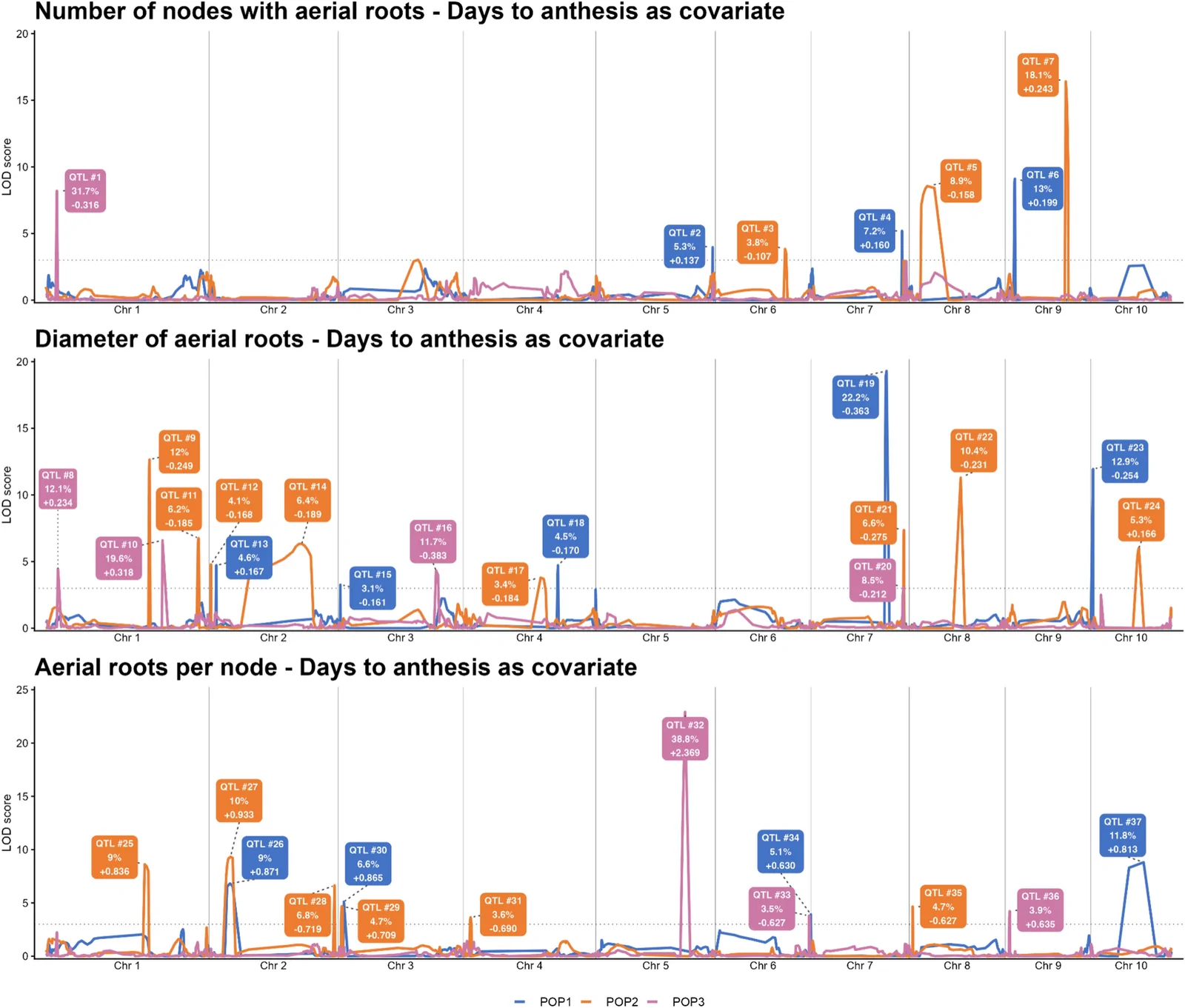
Genomic positions of 37 QTL candidates identified in populations 1 (blue), 2 (orange), and 3 (pink) for three aerial root traits, using days to anthesis as a covariate. The QTL positions are shown in the context of the B73 genome v5 assembly (Zm-B73-REFERENCE-NAM-5.0). On the x-axis, the 10 maize chromosomes are listed. The line charts (three per trait, one for each population) were plotted from the marker LOD scores, with the LOD scale on the y-axis. The dotted horizontal lines represent the LOD thresholds after 1,000 permutations. The QTL number/ID, the percentage of phenotypic variation explained (PVE%) by each QTL candidate, as well as its additive effect (positive or negative), is noted in the graphs, in balloons next to each QTL

Using the full dataset of three maize segregating populations (two BC_1_-based DH and one BC_1_-SSD_5_) across two locations (Georgia and Wisconsin) over two years (2023 and 2024), three, three, and one QTL were identified for the number of nodes with aerial roots on Pops 1, 2, and 3, respectively. These accounted for 23.4%, 29.5%, and 28.1% of the adjusted joint phenotypic variation (PVE_ADJ_), and additive effect sizes tended to be small, in the range of ±0.1 to ±0.3 nodes per QTL. Regarding aerial root diameter, five, eight, and four QTL were identified in the three populations evaluated, accounting for 46.3%, 50.7%, and 49.4% of the PVE_ADJ_, respectively; effect sizes ranged from ±0.2 to ±0.4 mm per QTL. For the number of aerial roots per node, four, six, and three QTL were identified on Pops 1, 2, and 3; these accounted for 31.4%, 37.3%, and 38.0% of the respective PVE_ADJ_, and additive effects ranged from ±0.6 to ±2.4 roots per node per QTL (Fig. 4, Supplementary Table ST12-ST13). Among the 37 QTL, 12 showed individual PVE > 10% (major QTL) and three of them showed individual PVE > 20%: the first in chromosome 1 (31.7% for nodes with aerial roots), the second on chromosome 5 (38.8% for the number of aerial roots per node), and the third on chromosome 7 (22.2% for the diameter of aerial roots).

There are two cases of QTL for different traits flanking each other, both on chromosome 1: QTL 1 (number of nodes with aerial roots) mapped just upstream of QTL 8 (aerial root diameter) in Pop 3, and QTL 25 (number of aerial roots per node) just upstream of QTL 9 (aerial root diameter) in Pop 2. Moreover, there are two cases of QTL colocalization across populations: on chromosome 2, QTL 26 (Pop 1) and 27 (Pop 2) mapped to the same region for the number of aerial roots per node; on chromosome 7, QTL 20 (Pop 3) and 21 (Pop 2) mapped to the same region for aerial root diameter. There is also one case of colocalization involving different traits: on chromosome 10, QTL 24 (mapped for aerial root diameter in Pop 2) overlaps with QTL 37 (mapped for the number of aerial roots per node in Pop 1) (Fig. 4, Supplementary Table ST12).

**Fig. 5:**
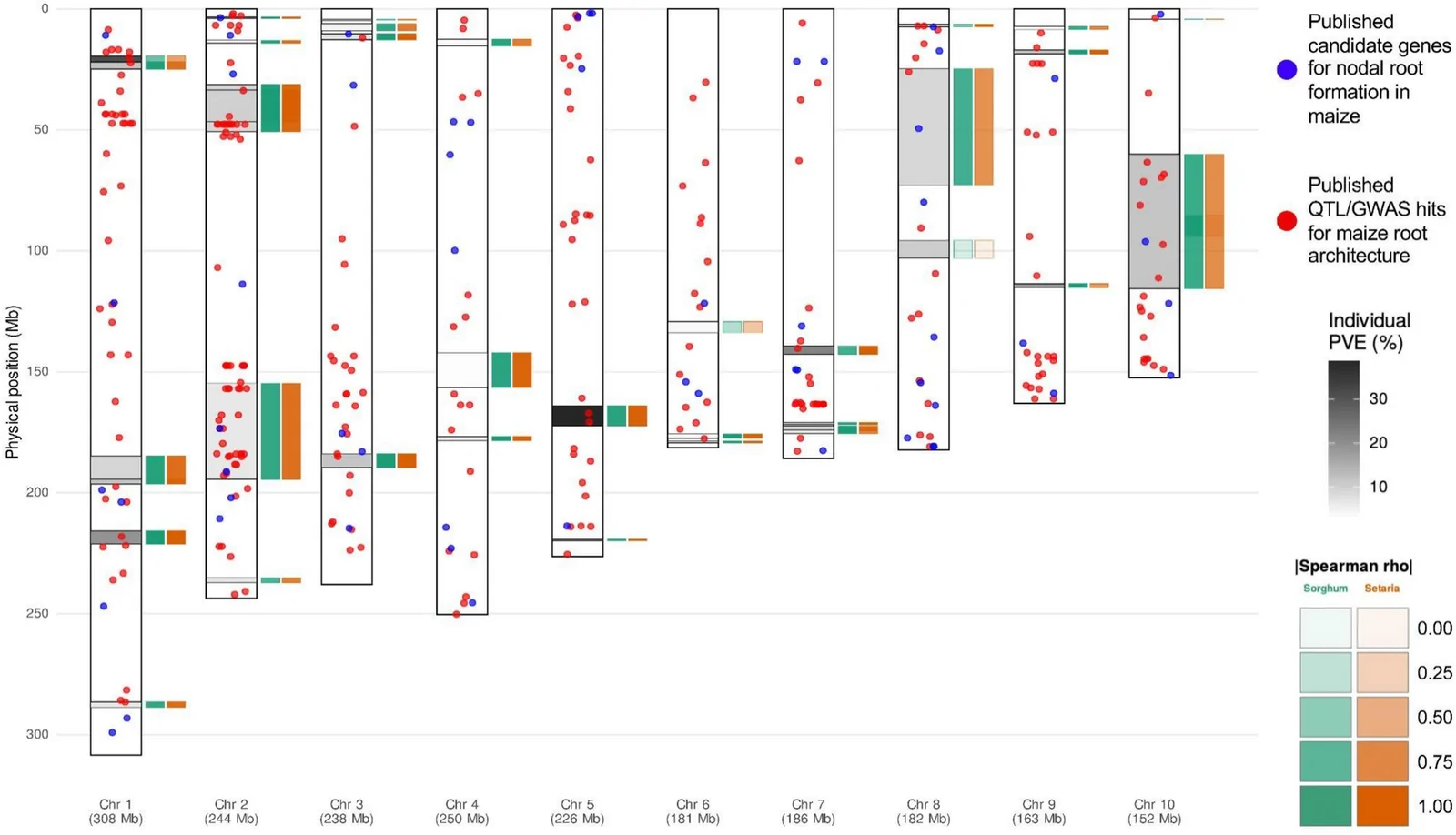
Overlap of 280 maize root architecture-associated published QTL/GWAS hits, 58 maize nodal root formation-associated annotated genes, and 37 aerial root-associated QTL mapped in this study, plus a summary of macrosynteny analyses. (i) The 280 QTL/GWAS hits (red dots) were compiled from twelve publications (Cai et al. 2012; Ku et al. 2012; Burton et al. 2014a, 2014b; Gu et al. 2017; Zhang et al. 2018; Wang et al. 2019; Sun et al. 2020, 2022; Moussa et al. 2021; Chen et al. 2023; Liu et al. 2023), where the following traits were assessed: root weight, root volume, root surface area, root angle, root branching number, root length, root number, root diameter, and number of nodes forming nodal roots, including crown (underground) and brace (aboveground) roots. (ii) The 58 genes (blue dots) involved in the formation of maize nodal roots were compiled from another twelve publications (Chen et al. 2021; Cowling et al. 2023; Hochholdinger et al. 2018; Hostetler et al. 2021; Li et al. 2019; Liu et al. 2022; Suzuki et al. 2015; Xu et al. 2015; Yang et al. 2022; Zhu et al. 2022; Zhang et al. 2015; Huang et al. 2017), and included maize orthologs of two genes associated with the number of nodes with aerial roots in sorghum (Wolf et al. 2024). (iii) The positions of the 37 mapped QTL from the current study are shown in the context of the B73 v5 genome (gray boxes), and the percentages of phenotypic variation explained by each one (individual PVE%) is indicated by shading. (iv) Macrosynteny comparisons of these QTL sorghum (green boxes) and foxtail millet (orange boxes) genomes are shown to the right of each QTL, with shading indicating the absolute values of Spearman’s rank correlation coefficient (|Spearman rho|), which indicates the collinearity strength

To identify potentially conserved regions, we performed a macrosynteny analysis against 2 other species with aerial roots, Sorghum (*Sorghum bicolor*) and foxtail millet (*Setaria italica*) (Sebastian et al. 2016; Hostetler et al. 2021; Tang et al. 2022; Sparks 2023). Sorghum showed slightly more collinear regions (30/37, 81.1%) over foxtail millet (28/37, 75.7%), as would be expected given its closer relationship to maize. Twenty-seven of the 37 QTL (73.0%) showed collinearity in both species, three were collinear in sorghum only, one in foxtail millet only, and six showed no evidence of collinearity in either species. Among the 58 collinear regions, gene order was strongly conserved (median |ρ|= 0.97; interquartile range 0.89–1.00), based on a median of 40 ortholog pairs per region (range 8–399), and orthologs were tightly concentrated on the expected target chromosome (median 87.6% of orthologs on the majority chromosome, meaning the one with the most orthologous hits). Of the collinear blocks, 33 (56.9%) retained the same gene order as maize (forward orientation) and 25 (43.1%) were inverted relative to maize (Fig. 5, Supplementary Table ST14).

### Candidate gene analysis

We compared the QTL intervals we mapped with 280 published QTL/GWAS hits for maize root morphology (Burton et al. 2014a; Burton et al. 2014b; Moussa et al. 2021; Sun et al. 2022; Sun et al. 2020; Ku et al. 2012; Wang et al. 2019; Gu et al. 2017; Cai et al. 2012; Zhang et al. 2018; Liu et al. 2023; Chen et al. 2023) and with 58 genes reportedly involved in the formation and growth of maize nodal roots (Chen et al. 2021; Cowling et al. 2023; Hochholdinger et al. 2018; Hostetler et al. 2021; Li et al. 2019; Liu et al. 2022; Suzuki et al. 2015; Xu et al. 2015; Yang et al. 2022; Zhu et al. 2022; Zhang et al. 2015; Huang et al. 2017); the latter included maize orthologs of two genes associated with the number of nodes with aerial roots in sorghum (Wolf et al. 2024) (Fig. 5). We found 56 overlapping hits and 6 overlapping genes: Zm00001eb067270, encoding the maize auxin response factor *ZmARF4*, which functions as a positive regulator of lateral root development and emergence (Cowling et al. 2023; Li et al. 2022); Zm00001eb122500, which corresponds to the Dwarf 1 (*ZmD1*) / *ZmGAOX3* gene encoding *Gibberellin 3-oxidase*, associated with changes in plant stature, brace root traits, nitrogen uptake, and nitrogen-use efficiency (Wang et al. 2025; Wang et al. 2020); Zm00001eb418700, known as *ZmCCT10* [*CO, CONSTANS, CO-like, and TIMING OF CAB1* (*CCT*) transcription factor (TF) family], a regulator of photoperiod response whose overexpression causes increased vegetative growth and the formation of secondary aerial roots (Stephenson et al. 2019; Zhong et al. 2026); Zm00001eb096740, a NAC-family TF that control lateral root development and is orthologous to genes involved in aerial root formation, elongation, and other root architectural changes via auxin-mediated responses in several species (Fan et al. 2014; Wolf et al. 2024); Zm00001eb100750, a bZIP TF known as *ZmABF2*, whose orthologs are involved in abscisic acid (ABA)-mediated aerial root formation and lateral root growth in response to nitrate availability (Guo et al. 2026; Wolf et al. 2024); and Zm00001eb341370, annotated as *Early phase change 1* (*ZmEpc1*), *Hasty 1* or *Exportin 5*, which heavily impacts axillary meristem initiation, leading to changes in vegetative phase change, leaf shape, shoot branching, tillering, and nodal root development (Jin et al. 2022; Sauer et al. 2023) (Supplementary Tables ST15-ST16).

When analyzing publicly available maize expression data (Stelpflug et al. 2016; Walley et al. 2016; Sekhon et al. 2012) on the qTeller platform (https://qteller.maizegdb.org/) (Woodhouse et al. 2021b), we observe that 5,007 genes located within the 37 mapped QTL exhibit highly variable expression in nodal roots and internode tissues (Supplementary Table ST17). After excluding low-expressed genes (ones with Fragments Per Kilobase of transcript per Million [FPKM] values <1 across three specific conditions), we see that 494 genes had their expressions downregulated (Log_2_ FC < − 1) in aerial roots from the first node above ground (called *brace roots from node 6* by Stelpflug et al. 2016) compared to tissues from the fourth elongated internode up to V9 phenological stage. Of these, 118 were strongly downregulated (Log_2_ FC < − 3). In this same comparison, 451 genes were upregulated on the aerial roots (Log_2_ FC > 1), of which 192 were strongly so (Log_2_ FC > 3) (Supplementary Fig S9A). A second comparison was made by analyzing the expression of these genes in the aforementioned aerial roots, originating from the first node above ground and at earlier stages of elongation and thickening, versus expression data from crown roots growing along the soil line, which were already fully elongated during phenological state V13. This time, 409 genes were downregulated (106 of them strongly) while 198 were upregulated (36 of them strongly) in the aerial roots (Supplementary Fig S9B). After noticing that 89 genes were substantially regulated (absolute Log_2_ FC > 2) across the two comparisons, we investigated their expression in nodal roots and internodes at various developmental stages (Supplementary Fig S10). 37 of these 89 differentially expressed genes (DEGs) grouped into four clusters: Cluster 1 (7 genes) is upregulated in aerial roots but downregulated in crown roots and internodes; Cluster 2 (8 genes) is the opposite (downregulated in aerial roots and upregulated in crown roots and internodes); Cluster 3 (17 genes) is upregulated in aerial roots and crown roots, but downregulated in internodes; and Cluster 4 (five genes) is opposite Cluster 3, being downregulated in aerial and crown roots but upregulated in internodes. The remaining 52 genes showed varied expression patterns across these three tissue groups.

## Discussion

The goal of this study was to identify QTL involved in aerial root formation in maize. Aerial root formation represents a critical first step for BNF to occur in maize, which ultimately involves a three-way interaction among the plant, the microbiome, and the environment (Van Deynze et al. 2018; Bennett et al. 2020; Chakraborty et al. 2024). Although all these components present opportunities for improvement, genetic improvement of the maize and/or microbial aspects are the most straightforward paths. (Adjusting the environment through, e.g., controlled irrigation is also an option but often represents a high infrastructure investment.)

### Characteristics of our mapping populations

Our mapping populations all derive from crosses between elite US-adapted materials and Oaxacan landraces with strong aerial root traits. Although the overall allele frequencies match expectations for BC_1_ populations (Supplementary Fig S3), a significant minority of markers in Pop 1 and Pop 2 (38.3% and 37.6%, respectively), deviated from the expected ratio at *p* < 0.05 (Supplementary Fig S5). These levels are consistent with published reports of segregation distortion in BC_1_-derived DH maize populations, where the selection pressures inherent in doubled haploid induction frequently result in departures from Mendelian expectations (Xu et al. 2013; Xu et al. 1997; Lu et al. 2002). Pop 3, in turn, showed considerably lower distortion, with only 10.2% of its markers reaching the *p* < 0.05 threshold (Supplementary Fig S5). Segregation Distortion Region (SDR) analysis identified nine putative SDRs in Pops 1 and 2 and one in Pop 3 (Supplementary Table ST5). These include a partially overlapping region on chromosome 3 in Pops 1 & 2 (23.7–104.2 cM and 71.7–122.3 cM, respectively), suggesting the potential presence of one or more segregation distortion loci here that operate independently of the specific Oaxacan donor parent. The single SDR in Pop 3 on chromosome 3 (107.4–125.7 cM, 10 markers, mean B73 frequency = 0.910) may reflect a region of genuine incompatibility or viability selection between the B73 and Olotón backgrounds. Consistent with established recommendations (Lyttle 1991; Vogl and Xu 2000), we kept all markers in the linkage maps to avoid creating large mapping gaps.

### Phenotypic variation, heritability, and trait correlations

In this study, our primary goal was to reveal the genetic contributions to three important aerial root traits potentially involved in BNF. First, the number of nodes with roots is important because each aerial root has a limited “lifespan” (that is, it secretes mucilage capable of hosting diazotrophs only for a certain period). Therefore, the formation and elongation of aerial roots on new nodes would extend the window for atmospheric nitrogen uptake. Second, aerial root diameter directly relates to the number of mucilage-producing border cells present in the aerial root tip and thus influences the quantity of mucilage that can be secreted. Third, the number of aerial roots per node also affects the total amount of mucilage that can potentially be produced, all other factors being equal (Van Deynze et al. 2018; Bennett et al. 2020; Pankievicz et al. 2022).

Our multi-year and multi-environment evaluations suggest aerial root traits have considerable environmental variability (Fig. 2). The phenotypic differences observed among populations and between locations are consistent with the contrasting genetic backgrounds of the three mapping populations and with the well-documented effects of photoperiod, temperature, and growing season length on maize vegetative development and aerial root architecture (Hostetler et al. 2021; Sparks 2023; Venado et al. 2025a). Wisconsin and Georgia represent markedly different growing environments: Georgia is characterized by longer growing seasons, higher average temperatures, greater humidity, and more intense solar radiation, while Wisconsin has longer photoperiods during the vegetative phase, conditions known to profoundly influence the rate of phytomer of production, internode elongation, and the timing of nodal root initiation (Song et al. 2016; Wen et al. 2021a, b; Lamb et al. 2024). Given that aerial root formation is minimally affected by soil nitrogen and ambient humidity (Venado et al. 2025a), other environmental factors (especially those related to light quantity and quality) should be investigated to elucidate which one is predominant in prompting the difference in the number of nodes with aerial roots observed at the two locations.

The fact that Pop 2 produced taller plants with thicker stalks (particularly in Georgia) suggests that its donor parent (Oaxaca 182) may contribute alleles that enhance vegetative growth under warm, long-season conditions. The contrasting behavior of Pop 1 and Pop 3, which consistently exhibited more nodes with aerial roots than Pop 2, may reflect alleles promoting nodal root bud initiation or outgrowth, traits that have been associated with auxin transport genes and specific transcription factors expressed during nodal root bud development (Xu et al. 2016; Omary et al. 2022; Singh et al. 2022). The tendency of Pop 2 to produce fewer nodes with aerial roots despite its greater overall vegetative vigor may indicate a trade-off between internode elongation and nodal root initiation, possibly mediated by differential auxin distribution (Strotmann and Stahl 2021; Lamb et al. 2024), but this needs to be explicitly tested. Root size tends to mirror that of the recurrent parents, with Pops 1 & 2 showing the thicker aerial roots characteristic of their common parent, PHZ51, whereas Pop 3 generally exhibits the thinner roots of its parent B73. The location-by-population interaction observed for the number of aerial roots per node (where Pops 1 and 3 were statistically different from Pop 2 in Wisconsin but not in Georgia) suggests significant genotype-by-environment (G×E) interaction, as has been reported for root architectural traits in maize (Viana et al. 2022). Finally, the consistently higher values for aerial root diameter, aerial roots per node, and stalk diameter observed in 2023 compared to 2024 across both locations (Fig. 2) suggest year-to-year environmental variation is also a significant phenotypic driver,and that more environments will be necessary to better understand G×E interactions for aerial root traits (Hudson et al. 2022). Despite these environmental influences, aerial root traits still exhibit moderately high heritability, with H^2^ ranging from 0.65 to 0.83 (Fig. 3A), indicating significant potential for directional selection in breeding programs. The magnitude and consistency of the gap between broad- (H^2^) and narrow (h^2^_SNP_)-sense heritability across different traits and populations, meanwhile, is also very informative. For example, in traits such as flag leaf height, where H^2^ was high and consistent between populations (0.9) but h^2^_SNP_ was low-to-moderate (0.46 and 0.36 for Pops 1 and 2, respectively), the divergence suggests that a substantial share of the genetic variance in these populations is not well tagged by the current marker density; this is consistent with a highly polygenic architecture with many small-effect genes, as previously reported (Peiffer et al. 2014; Wallace et al. 2016; Anderson et al. 2020; Michel et al. 2022), although this may also occur due to confounding factors such as common environmental effects and non-additive genetic variance (Yang, J et al. 2017). As such, these traits will be difficult to resolve to individual QTL loci and may be better addressed through polygenic or genomic prediction frameworks, which can capture missing variance by treating all marker effects as random and aggregating minor effects (Bian and Holland 2017). In contrast, traits with closer agreement between the two heritability estimates, such as aerial root diameter (H^2^ up to 0.83 and h^2^_SNP_ up to 0.64) and number of nodes with aerial roots (H^2^ up to 0.78 and h^2^_SNP_ up to 0.54), are more promising candidates for identifying individual loci with relevant effect (Su et al. 2015).

### Choice of covariate for residualization

We considered three potential covariates for the aerial root traits in our analysis: (i) Stalk diameter, since larger stalks could accommodate more roots; (ii) flowering time (days to anthesis) since plants with longer vegetative growth would have more time to form aerial roots; and (iii) plant height (flag leaf height), which tends to be correlated with both previous traits. Each of these traits showed significant correlations to aerial root traits (Fig. 3B), though the degree and significance varied by trait. Ultimately, we included days to anthesis as a covariate in our statistical models because of its importance for overall maize development. Trait residuals after accounting for anthesis were more normally distributed than the raw data, so its removal also produced distributions more compatible with the normality assumptions of QTL mapping models (Supplementary Fig S6-S8). Although some traits still showed significant non-normality after residualization, IciMapping generally proves robust to moderate deviations from normality of this type, particularly with sample sizes above 80 (Meng et al. 2015).

### Mapped QTL can aid in maize breeding efforts for increasing BNF

We mapped 37 QTL for aerial root traits with data spanning two years in two contrasting environments. These QTL explain a combined 23 to 51% of the phenotypic variance observed for each trait. Among them, 12 can be considered major QTL, as they individually explained more than 10% of the phenotypic variance (Fig. 4, Supplementary Table ST12). Three of them (QTL 1, 19, and 32) show promise for future marker-assisted selection steps in breeding programs due to their large PVE (31.7% for nodes with aerial roots in Pop 3, 22.2% for aerial root diameter in Pop 1, and 38.8% for the number of aerial roots per node in Pop 3). However, since only one location’s data is available for Pop 3, its results will need to be validated over a wider geographic range.

One might expect that multiple QTL should be colocalized across our three populations, instead of just the two we identified (QTL 26 from Pop 1 and QTL 27 from Pop 2 on chromosome 2 for the number of aerial roots per node; QTL 20 from Pop 3 and QTL 21 from Pop 2 on chromosome 7 for aerial root diameter). However, these populations had different donor parents and are likely segregating for different alleles; in addition, different mapping populations may capture a causal mutation through different flanking markers so that two distinct QTL may be tagging the same underlying gene through different linkage disequilibrium (LD) blocks. Similarly, different recombination events can cause QTL intervals to shift because of different haplotype boundaries in each population. For these reasons, mining these QTL regions for candidate genes, rather than assuming precise QTL positions, would be the most appropriate strategy for follow-up projects.

Macrosynteny analyses were performed to examine medium-scale chromosomal conservation for the 37 mapped QTL across other grasses where aerial roots have been observed (Sebastian et al. 2016; Hostetler et al. 2021; Tang et al. 2022; Sparks 2023). High levels of collinearity in sorghum (81.1%) and foxtail millet (75.7%), as well as the gene order (56.9% retained the forward orientation) (Fig. 5, Supplementary Table ST16) are consistent with local structural rearrangements accumulated since these species diverged from a common ancestor (Guillotin et al. 2023). These results indicate that most maize QTL intervals identified in this study retain substantial macrosynteny with sorghum and foxtail millet, supporting the use of comparative genomics to prioritize candidate genes within those regions. Nevertheless, targeted follow-up studies will be needed to confirm the roles of potential candidate genes in aerial root morphology in all three grasses.

### Identification of candidate genes for traits related to aerial root formation

Both brace and aerial roots originate from stem nodes through a five-step process: initiation, outgrowth, emergence, elongation, and thickening. This process represents a tightly coordinated set of timed transitions, hormone signaling, and nutrient sensing (Hostetler et al. 2021; Jan et al. 2024; Singh et al. 2022; Xu et al. 2016; Omary et al. 2022). One would thus expect many genes–and therefore many QTL–to be involved in each of the three traits. To identify potential candidate genes, we compared our results to studies involving the formation & growth of maize nodal roots (Chen et al. 2021; Cowling et al. 2023; Hochholdinger et al. 2018; Hostetler et al. 2021; Li et al. 2019; Liu et al. 2022; Suzuki et al. 2015; Xu et al. 2015; Yang et al. 2022; Zhu et al. 2022; Zhang et al. 2015; Huang et al. 2017; Wolf et al. 2024) (Supplementary Table ST15). Our findings highlighted six gene models whose functions make them likely candidate genes. However, confirmation of their potential rolls will require more targeted analyses (e.g., single-cell RNA-seq, spatial transcriptomics).

Given that the aerial root traits studied here derive from specific Oaxacan landraces that have yet to be sequenced, it will be necessary to sequence and annotate their genes in the most promising QTL to facilitate functional prediction and validation of these associations. It should also be noted that given the large gene space covered by QTL we mapped, one would expect some overlap with any candidate gene list by chance alone. As such, these should be taken as preliminary associations that will require higher-resolution follow-up analyses to confirm.

We also performed an in-silico expression analysis of candidate genes by mining public gene expression datasets, targeting gene expression in the genes located on the 37 QTL intervals we had identified (Supplementary Table ST17). Comparing early aerial root growth against fully elongated crown roots and against internodes identified hundreds of DEGs (Supplementary Fig S9). Eighty-nine genes that were significantly up- or down-regulated in both comparisons were examined in more detail, then clustered into groups with similar expression patterns (Supplementary Fig S10). Members of the first gene cluster appear promising due to their high expression in aerial roots at earlier stages of development, combined with the low-to-medium expression in crown roots and internodes. This expression profile suggests that they could be involved in aerial root elongation or thickening; microscopic analysis during the establishment of founder cells (FCs) would be required to determine if they play a role earlier (during root initiation). Taken together, these insights on candidate genes may guide validation projects and, in the future,assist in introgressing aerial root traits into elite materials. Complementary studies should be prioritized to further identify key genes in aerial root development and link molecular activity to tissue structure (Yin et al. 2023; Yang et al. 2025).

### Prospects for nitrogen fixation in temperate maize

The results presented here indicate promise for introgressing aerial root traits associated with biological nitrogen fixation into temperate maize. Heritability estimates and the identification of genomic loci associated with these traits strongly suggest that the aerial root phenotype can be selected for and improved in temperate maize.

The findings of the present study must be interpreted within the rapidly expanding landscape of aerial root- and mucilage-mediated BNF across plant species. When Van Deynze et al. (2018) first demonstrated that Oaxacan maize landraces support a mucilage-associated diazotrophic microbiota, this system was largely considered a curiosity specific to a narrow set of landraces under specific growing conditions. The subsequent years have substantially revised this view. Pankievicz et al. (2022) established that mucilage production and border cell function are the primary determinants of diazotrophic community assembly on maize aerial roots, decoupling nitrogen fixation capacity from the specific landrace background and pointing toward portable genetic determinants of the trait. Venado et al. (2025b) extended this framework to sorghum, demonstrating that mucilage produced by sorghum aerial roots hosts nitrogen-fixing diazotrophs that provide measurable nitrogen to the plant, a finding with major implications for the generality of the grass aerial root BNF system. Wolf et al. (2024) identified genetic and environmental factors influencing aerial root traits associated with BNF in sorghum, providing a comparative QTL framework that partially overlaps with chromosomal regions identified in the present study in maize. Taken together, these parallel developments position the aerial root mucilage BNF system not as an isolated curiosity but as one manifestation of a broader biological principle: aerial root-produced mucilage provides a carbon-rich microhabitat that selectively enriches diazotrophic bacteria and supports nitrogen fixation. The QTL identified in the present study for nodes with aerial roots, aerial root diameter, and aerial roots per node therefore represent not only markers for potentially improving nitrogen fixation capacity in maize specifically, but also candidate genomic regions for comparative mapping across grass species to identify conserved aspects of aerial root architecture development.

Several challenges and open questions must be addressed to fully harness aerial roots for BNF, including water requirements, reliance on diazotrophic microbes, and potential yield reductions due to metabolic investment in aerial roots and mucilage. The net agronomic value of this system depends critically on whether the nitrogen returned to the plant via BNF is sufficient to compensate for the metabolic cost of maintaining this system. Current rough estimates for maize suggest a potential yield penalty associated with this carbon investment of approximately 2–11%, depending on genotype, environmental conditions, and the specific nitrogen economy of the system (Van Gelder et al. 2023). This range is wide enough to encompass scenarios where BNF is clearly net beneficial as well as ones where it is not. Whether the trade-off is agronomically worthwhile will ultimately depend on factors that vary substantially across production systems, especially the price of nitrogen fertilizer relative to grain price, the soil nitrogen status of the field, and the degree to which synthetic nitrogen inputs can be reduced without yield penalty when BNF is active. In low-input or organic production systems where nitrogen fertilizer is expensive or unavailable, even a modest BNF contribution could more than offset a minor yield penalty, making the trait strongly net positive. In high-input systems where nitrogen is cheap and abundant, the same tradeoff would be harder to justify economically. These considerations argue for the development of genotypes in which aerial root architecture and mucilage production are optimized to maximize nitrogen fixation efficiency per unit of carbon invested, rather than simply maximizing aerial root number or biomass. The QTL framework developed in the present study may help to address this breeding objective by identifying genomic regions controlling key components of aerial root architecture.

## Supplementary Information

Below is the link to the electronic supplementary material.

Supplementary file1 (DOCX 3792 KB)

Supplementary file2 (XLSX 6051 KB)

## Data Availability

The data (genotypes and phenotypes) used in this study are available as supplementary material on the publisher’s website (Supplemental File 2) and in the linked Github repository (https://github.com/wallacelab/paper-laspisa-aerial-root-qtl-2026).

## References

[CR1] Anderson SL 2nd et al (2020) Unoccupied aerial system enabled functional modeling of maize height reveals dynamic expression of loci. Plant Direct 4(5):E00223. 10.1002/pld3.223 32399510 PMC7212003

[CR2] Bates D et al (2015) Fitting linear mixed-effects models using lme4. J Stat Softw 67(1):1–48. 10.18637/jss.v067.i01

[CR3] Bennett AB, Pankievicz VCS, Ané J-M (2020) A model for nitrogen fixation in cereal crops. Trends Plant Sci 25(3):226–235. 10.1016/j.tplants.2019.12.004 31954615

[CR4] Bian Y, Holland J (2017) Enhancing genomic prediction with genome-wide association studies in multiparental maize populations. Heredity Edinb 118:585–593. 10.1038/hdy.2017.4 28198815 PMC5436027

[CR5] Bradbury PJ, Zhang Z, Kroon DE, Casstevens TM, Ramdoss Y, Buckler ES (2007) TASSEL: software for association mapping of complex traits in diverse samples. Bioinformatics 23(19):2633–2635. 10.1093/bioinformatics/btm308 17586829

[CR6] Burton AL et al (2014a) QTL mapping and phenotypic variation for root architectural traits in maize (*Zea mays* L.). Theor Appl Genet 127(11):2293–2311. 10.1007/s00122-014-2353-4 25230896

[CR7] Burton AL et al (2014b) QTL mapping and phenotypic variation of root anatomical traits in maize (*Zea mays* L.). Theor Appl Genet 128(1):93–106 25326723 10.1007/s00122-014-2414-8

[CR8] Cai H et al (2012) Identification of QTLs for plant height, ear height and grain yield in maize (*Zea mays* L.) in response to nitrogen and phosphorus supply. Plant Breed 131(4):502–510. 10.1111/j.1439-0523.2012.01963.x

[CR9] Camargo JA, Alonso Á (2006) Ecological and toxicological effects of inorganic nitrogen pollution in aquatic ecosystems: a global assessment. Environ Int 32(6):831–849. 10.1016/j.envint.2006.05.002 16781774

[CR10] Chakraborty S et al (2024) Scripting a new dialogue between diazotrophs and crops. Trends Microbiol 32(6):577–589. 10.1016/j.tim.2023.08.007 37770375 PMC10950843

[CR11] Chen G et al (2021) Gravity reduced nitrogen uptake via the regulation of brace unilateral root growth in maize intercropping. Front Plant Sci. 10.3389/fpls.2021.724909 34552608 PMC8450519

[CR12] Chen G et al (2023) Genetic basis of resistance to southern corn leaf blight in the maize multi-parent population and diversity panel. Plant Biotechnol J 21(3):506–520. 10.1111/pbi.13967 36383026 PMC9946143

[CR13] Cowling CL, Dash L, Kelley DR (2023) Roles of auxin pathways in maize biology. J Exp Bot 74(22):6989–6999. 10.1093/jxb/erad297 37493143 PMC10690729

[CR14] Dixon R, Kahn D (2004) Genetic regulation of biological nitrogen fixation. Nat Rev Microbiol 2(8):621–631. 10.1038/nrmicro954 15263897

[CR15] Dobin A, Gingeras TR (2015) Mapping RNA-seq reads with STAR. Curr Protoc Bioinformatics 51(1):11.14.1-11.14.19. 10.1002/0471250953.bi1114s51 26334920 PMC4631051

[CR16] Edet OU et al (2018) DArTseq-based analysis of genomic relationships among species of tribe Triticeae. Sci Rep 8(1):16397 30401925 10.1038/s41598-018-34811-y PMC6219600

[CR17] Endelman JB (2011) Ridge regression and other kernels for genomic selection with R package rrBLUP. Plant Genome 4(3):250–255. 10.3835/plantgenome2011.08.0024

[CR18] Erenstein O et al (2022) Global maize production, consumption and trade: trends and R&D implications. Food Security 14(5):1295–1319. 10.1007/s12571-022-01288-7

[CR19] Fan K et al (2014) Molecular evolution and expansion analysis of the NAC transcription factor in *Zea mays*. PLoS ONE 9(11):e111837. 10.1371/journal.pone.0111837 25369196 PMC4219692

[CR20] Goodkind AL et al (2023) Managing nitrogen in maize production for societal gain. PNAS Nexus 2(10):gad319. 10.1093/pnasnexus/pgad319 PMC10597588 37881340

[CR21] Goodstein DM et al (2012) Phytozome: a comparative platform for green plant genomics. Nucleic Acids Res 40(D1):D1178–D1186. 10.1093/nar/gkr944 22110026 PMC3245001

[CR22] Gu D et al (2017) QTL identification for brace-root traits of maize in different generations and environments. Crop Sci 57(1):13–21. 10.2135/cropsci2016.01.0031

[CR23] Guillotin B, Rahni R, Passalacqua M et al (2023) A pan-grass transcriptome reveals patterns of cellular divergence in crops. Nature 617:785–791. 10.1038/s41586-023-06053-0 37165193 PMC10657638

[CR24] Guo Z et al (2021) Identification of major QTL for waterlogging tolerance in maize using genome-wide association study and bulked sample analysis. J Appl Genet 62(3):405–418. 10.1007/s13353-021-00629-0 33788096

[CR25] Guo K et al (2023) Biological nitrogen fixation in cereal crops: progress, strategies, and perspectives. Plant Commun 4(2):100499. 10.1016/j.xplc.2022.100499 36447432 PMC10030364

[CR26] Guo Z, Gao X, Wang C, Liu Z, Chen Z, Ning S, Xiao M, Fei J, Zhang Q (2026) Genome-wide identification and functional characterization of the ABF gene family in maize reveals ZmABF8 as a key regulator of drought tolerance. Front Plant Sci. 10.3389/fpls.2026.1849922 42255300 PMC13233282

[CR27] Hochholdinger F, Yu P, Marcon C (2018) Genetic control of root system development in maize. Trends Plant Sci 23(1):79–88. 10.1016/j.tplants.2017.10.004 29170008

[CR28] Holland J, Nyquist WE, Cervantes-Martinez CT (2003) Estimating and interpreting heritability for plant breeding: an update. Plant Breeding Reviews 22:9–111

[CR29] Holland JB, Piepho H-P (2024) Don't BLUP twice. G3 Bethesda, Md 14(12):jkae250 10.1093/g3journal/jkae250 PMC11631391 39558791

[CR30] Hostetler AN et al (2021) Bracing for sustainable agriculture: the development and function of brace roots in members of Poaceae. Curr Opin Plant Biol 59:101985. 10.1016/j.pbi.2020.101985 33418403

[CR31] Huang P et al (2017) Sparse panicle1 is required for inflorescence development in *Setaria viridis* and maize. Nat Plants 3(5):17054. 10.1038/nplants.2017.54 28418381

[CR32] Hudson AI et al (2022) Analysis of genotype-by-environment interactions in a maize mapping population. G3 Genes Genomes Genet 12(3):jkac013. 10.1093/g3journal/jkac013 PMC8895993 35134181

[CR33] Hufford MB et al (2021) De novo assembly, annotation, and comparative analysis of 26 diverse maize genomes. Science 373(6555):655–662. 10.1126/science.abg5289 34353948 PMC8733867

[CR34] Inoue T, Kohzu A, Akaji Y, Miura S, Baba S (2024) Diazotrophic nitrogen fixation through aerial roots occurs in *Avicennia marina*: implications for adaptation of mangrove plant growth to low-nitrogen tidal flats. New Phytol 241:1464–1475. 10.1111/nph.19442 38013587

[CR35] Jan M, Muhammad S, Jin W, Zhong W, Zhang S, Lin Y, Zhou Y, Liu J, Liu H, Munir R, Yue Q, Afzal M, Wang G (2024) Modulating root system architecture: cross-talk between auxin and phytohormones. Front Plant Sci 15:1343928. 10.3389/fpls.2024.1343928 38390293 PMC10881875

[CR36] Jin L, Zhang G, Yang G, Dong J (2022) Identification of the Karyopherin superfamily in maize and its functional cues in plant development. Int J Mol Sci 23(22):14103. 10.3390/ijms232214103 36430578 PMC9699179

[CR37] Ku LX et al (2012) QTL mapping and epistasis analysis of brace root traits in maize. Mol Breed 30(2):697–708. 10.1007/s11032-011-9655-x

[CR38] Lamb A et al (2024) Bioenergy sorghum nodal root bud development: morphometric, transcriptomic and gene regulatory network analysis. Front Plant Sci. 10.3389/fpls.2024.1456627 39498396 PMC11532172

[CR39] Lawrence M, Gentleman R, Carey V (2009) Rtracklayer: an R package for interfacing with genome browsers. Bioinformatics 25(14):1841–1842. 10.1093/bioinformatics/btp328 19468054 PMC2705236

[CR40] Lenth RV (2024) emmeans: estimated marginal means, aka least-squares means. R package. https://cran.r-project.org/package=emmeans

[CR41] Li J et al (2019) *ZmRAP2.7*, an AP2 transcription factor, is involved in maize brace roots development. Front Plant Sci. 10.3389/fpls.2019.00820 31333689 PMC6621205

[CR42] Li J et al (2022) Maize transcription factor ZmARF4 confers phosphorus tolerance by promoting root morphological development. Int J Mol Sci. 10.3390/ijms23042361 35216479 PMC8880536

[CR43] Lin S et al (2024) Genome-wide association study and genomic selection of brace root traits related to lodging resistance in maize. Sci Rep 14(1):31898. 10.1038/s41598-024-83230-9 39738690 PMC11686075

[CR44] Liu M et al (2022) A genome-wide association study dissects the genetic architecture of the metaxylem vessel number in maize brace roots. Front Plant Sci 13:847234. 10.3389/fpls.2022.847234 35360304 PMC8961028

[CR45] Liu Z et al (2023) Hybrid performance evaluation and genome-wide association analysis of root system architecture in a maize association population. Theor Appl Genet 136(9):194. 10.1007/s00122-023-04442-7 37606710

[CR46] Liu X et al (2024) Impact of groundwater nitrogen legacy on water quality. Nat Sustain 7(7):891–900. 10.1038/s41893-024-01369-9

[CR47] Lu H, Romero-Severson J, Bernardo R (2002) Chromosomal regions associated with segregation distortion in maize. Theor Appl Genet 105(4):622–628. 10.1007/s00122-002-0970-9 12582513

[CR48] Lyttle TW (1991) Segregation distorters. Annu Rev Genet 25:511–581. 10.1146/annurev.ge.25.120191.002455 1812815

[CR49] Meng L et al (2015) QTL IciMapping: integrated software for genetic linkage map construction and quantitative trait locus mapping in biparental populations. Crop J 3(3):269–283. 10.1016/j.cj.2015.01.001

[CR50] Michel KJ et al (2022) Genetic mapping and prediction of flowering time and plant height in a maize Stiff Stalk MAGIC population. Genetics. 10.1093/genetics/iyac063 35441688 PMC9157087

[CR51] Moussa AA et al (2021) Genome-wide association screening and verification of potential genes associated with root architectural traits in maize (Zea mays L.) at multiple seedling stages. BMC Genomics 22(1):558. 10.1186/s12864-021-07874-x 34284723 PMC8290564

[CR52] Omary M et al (2022) A conserved superlocus regulates above- and belowground root initiation. Science 375(6584):eabf4368. 10.1126/science.abf4368 35239373

[CR53] Pan S-Y et al (2022) Addressing nitrogenous gases from croplands toward low-emission agriculture. Npj Climate Atmos Sci. 10.1038/s41612-022-00265-3

[CR54] Pankievicz VCS et al (2022) Nitrogen fixation and mucilage production on maize aerial roots is controlled by aerial root development and border cell functions. Front Plant Sci 13:977056. 10.3389/fpls.2022.977056 36275546 PMC9583020

[CR55] Peiffer JA et al (2014) The genetic architecture of maize height. Genetics 196(4):1337–1356. 10.1534/genetics.113.159152 24514905 PMC3982682

[CR56] Piepho H-P, Möhring J (2007) Computing heritability and selection response from unbalanced plant breeding trials. Genetics 177:1881–1888. 10.1534/genetics.107.074229 18039886 PMC2147938

[CR57] R Core Team (2024) R: a language and environment for statistical computing (version 4.4.0). Computer software

[CR58] Sauer M, Zhao J, Park M, Khangura RS, Dilkes BP, Poethig RS (2023) Identification of the Teopod1, Teopod2, and early phase change genes in maize. G3 (Bethesda, Md.) 13(10):jkad179 10.1093/g3journal/jkad179 PMC10542106 37548268

[CR59] Sebastian J et al (2016) Grasses suppress shoot-borne roots to conserve water during drought. Proc Natl Acad Sci U S A 113(31):8861–8866. 10.1073/pnas.1604021113 27422554 PMC4978293

[CR60] Sekhon RS et al (2012) Transcriptional and metabolic analysis of senescence induced by preventing pollination in maize. Plant Physiol 159(4):1730–1744. 10.1104/pp.112.199224 22732243 PMC3425209

[CR61] Singh Z et al (2022) Genetic and hormonal blueprint of shoot-borne adventitious root development in rice and maize. Plant Cell Physiol 63(12):1806–1813. 10.1093/pcp/pcac084 35713294

[CR62] Snapp S et al (2023) Spatially differentiated nitrogen supply is key in a global food–fertilizer price crisis. Nat Sustain 6(10):1268–1278. 10.1038/s41893-023-01166-w

[CR63] Song Y et al (2016) Morphological characteristics of maize canopy development as affected by increased plant density. PLoS ONE 11(4):e0154084. 10.1371/journal.pone.0154084 27129101 PMC4851380

[CR64] Sparks EE (2023) Maize plants and the brace roots that support them. New Phytol 237(1):48–52. 10.1111/nph.18489 36102037

[CR65] Stelpflug SC et al (2016) An expanded maize gene expression atlas based on RNA sequencing and its use to explore root development. Plant Genome. 10.3835/plantgenome2015.04.0025 27898762

[CR66] Stephenson E, Estrada S, Meng X, Ourada J, Muszynski MG, Habben JE, Danilevskaya ON (2019) Over-expression of the photoperiod response regulator ZmCCT10 modifies plant architecture, flowering time and inflorescence morphology in maize. PLoS ONE 14(2):e0203728. 10.1371/journal.pone.0203728 30726207 PMC6364868

[CR67] Strotmann VI, Stahl Y (2021) At the root of quiescence: function and regulation of the quiescent center. J Exp Bot 72(19):6716–6726. 10.1093/jxb/erab275 34111273

[CR68] Su CF et al (2015) Influence of various quantitative trait loci (QTL) mapping methods on the mapping accuracy under varying heritability levels. Genet Mol Res 14(4):13003–13012. 10.4238/2015.October.21.21 26505453

[CR69] Sun N et al (2020) QTL identification in backcross population for brace-root-related traits in maize. Euphytica 216(2):32. 10.1007/s10681-020-2561-8

[CR70] Sun D et al (2022) Genome-wide association study reveals the genetic basis of brace root angle and diameter in maize. Front Genet 13:963852. 10.3389/fgene.2022.963852 36276979 PMC9582141

[CR71] Suzuki M et al (2015) Conserved functions of the MATE transporter BIG EMBRYO1 in regulation of lateral organ size and initiation rate. Plant Cell 27(8):2288–2300. 10.1105/tpc.15.00290 26276834 PMC4568504

[CR72] Swarts K et al (2014) Novel methods to optimize genotypic imputation for low-coverage, next-generation sequence data in crop plants. Plant Genome. 10.3835/plantgenome2014.05.0023

[CR73] Tang S et al (2022) The role of AUX1 during lateral root development in the domestication of the model C4 grass *Setaria italica*. J Exp Bot 73(7):2021–2034. 10.1093/jxb/erab556 34940828

[CR74] Trapnell C et al (2012) Differential gene and transcript expression analysis of RNA-seq experiments with TopHat and Cufflinks. Nat Protoc 7(3):562–578. 10.1038/nprot.2012.016 22383036 PMC3334321

[CR75] USDA (2024) World agricultural production. Circular Series WAP 12–24. United States Department of Agriculture, Washington, DC. https://apps.fas.usda.gov/psdonline/circulars/production.pdf

[CR76] Van Deynze A et al (2018) Nitrogen fixation in a landrace of maize is supported by a mucilage-associated diazotrophic microbiota. PLoS Biol 16(8):e2006352. 10.1371/journal.pbio.2006352 30086128 PMC6080747

[CR77] Van Gelder K et al (2023) Running the numbers on plant synthetic biology solutions to global problems. Plant Sci 335:111815. 10.1016/j.plantsci.2023.111815 37543223

[CR78] Venado RE et al (2025a) Aerial root formation in Oaxacan maize (Zea mays) landraces persists into the adult phase and is minimally affected by soil nitrogen and ambient humidity. Front Plant Sci. 10.3389/fpls.2025.1607733 40718026 PMC12289584

[CR79] Venado RE et al (2025b) Mucilage produced by aerial roots hosts diazotrophs that provide nitrogen in *Sorghum bicolor*. PLoS Biol 23(3):e3003037. 10.1371/journal.pbio.3003037 40029899 PMC12136154

[CR80] Viana WG, Scharwies JD, Dinneny JR (2022) Deconstructing the root system of grasses through an exploration of development, anatomy and function. Plant Cell Environ 45(3):602–619. 10.1111/pce.14270 35092025 PMC9303260

[CR81] Vogl C, Xu S (2000) Multipoint mapping of viability and segregation distorting loci using molecular markers. Genetics 155(3):1439–1447. 10.1093/genetics/155.3.1439 10880501 PMC1461139

[CR82] Vos R et al (2025) Global shocks to fertilizer markets: impacts on prices, demand and farm profitability. Food Policy 133:102790. 10.1016/j.foodpol.2024.102790

[CR83] Wallace JG et al (2016) Genome-wide association for plant height and flowering time across 15 tropical maize populations under managed drought stress and well-watered conditions in Sub-Saharan Africa. Crop Sci 56(5):2365–2378. 10.2135/cropsci2015.10.0632

[CR84] Walley JW et al (2016) Integration of omic networks in a developmental atlas of maize. Science 353(6301):814–818. 10.1126/science.aag1125 27540173 PMC5808982

[CR85] Wang H et al (2019) Integrating GWAS and gene expression analysis identifies candidate genes for root morphology traits in maize at the seedling stage. Genes. 10.3390/genes10100773 31581635 PMC6826382

[CR86] Wang Y et al (2020) The role of gibberellins in regulation of nitrogen uptake and physiological traits in maize responding to nitrogen availability. Int J Mol Sci 21(5):1824. 10.3390/ijms21051824 32155833 PMC7084584

[CR87] Wang P, Liang B, Li Z, Dong H, Zhang L, Lu X (2025) The identification of a single-base mutation in the maize dwarf 1 gene responsible for reduced plant height in the mutant 16N125. Plants (Basel) 14(8):1217. 10.3390/plants14081217 40284105 PMC12030145

[CR88] Wen A et al (2021a) Enabling biological nitrogen fixation for cereal crops in fertilized fields. ACS Synth Biol 10(12):3264–3277. 10.1021/acssynbio.1c00049 34851109

[CR89] Wen W et al (2021b) 3D phytomer-based geometric modelling method for plants – the case of maize. AoB Plants 13(5):lab055. 10.1093/aobpla/plab055 PMC8482417 34603653

[CR90] Wickham H (2016) Ggplot2: elegant graphics for data analysis. Springer-Verlag, New York

[CR91] Wolf ESA et al (2024) Identification of genetic and environmental factors influencing aerial root traits that support biological nitrogen fixation in sorghum. G3: Genes, Genomes, Genet. 10.1093/g3journal/jkad285 PMC10917507 38096484

[CR92] Woodhouse MR et al (2021a) A pan-genomic approach to genome databases using maize as a model system. BMC Plant Biol 21(1):385. 10.1186/s12870-021-03173-5 34416864 PMC8377966

[CR93] Woodhouse MR et al (2021b) qTeller: a tool for comparative multi-genomic gene expression analysis. Bioinformatics 38(1):236–242. 10.1093/bioinformatics/btab604 34406385

[CR94] Xu Y et al (1997) Chromosomal regions associated with segregation distortion of molecular markers in F2, backcross, doubled haploid, and recombinant inbred populations in rice (*Oryza sativa* L.). Mol Gen Genet 253(5):535–545. 10.1007/s004380050355 9065686

[CR95] Xu X et al (2013) Gametophytic and zygotic selection leads to segregation distortion through in vivo induction of a maternal haploid in maize. J Exp Bot 64(4):1083–1096. 10.1093/jxb/ers393 23349137 PMC3580820

[CR96] Xu C et al (2015) Cooperative action of the paralogous maize lateral organ boundaries domain proteins RTCS and RTCL in shoot-borne root formation. New Phytol 207(4):1123–1133. 10.1111/nph.13420 25902765

[CR97] Xu C, Luo F, Hochholdinger F (2016) LOB domain proteins: beyond lateral organ boundaries. Trends Plant Sci 21(2):159–167. 10.1016/j.tplants.2015.10.010 26616195

[CR98] Yang J et al (2017) Concepts, estimation and interpretation of SNP-based heritability. Nat Genet 49:1304–1310. 10.1038/ng.3941 28854176

[CR99] Yang F et al (2022) ZmIAA5 regulates maize root growth and development by interacting with ZmARF5 under the specific binding of ZmTCP15/16/17. PeerJ 10:e13710. 10.7717/peerj.13710 35855434 PMC9288822

[CR100] Yang Y-J et al (2025) Spatial transcriptomics provide insights into the secondary lateral rooting difficulties of tetraploid forsythia cuttings. Ind Crops Prod 237:122182. 10.1016/j.indcrop.2025.122182

[CR101] Yin R, Xia K, Xu X (2023) Spatial transcriptomics drives a new era in plant research. Plant J 116(6):1571–1581. 10.1111/tpj.16437 37651723

[CR102] Zhang Y et al (2015) LATERAL ROOT PRIMORDIA 1 of maize acts as a transcriptional activator in auxin signalling downstream of the Aux/IAA gene rootless with undetectable meristem 1. J Exp Bot 66(13):3855–3863. 10.1093/jxb/erv187 25911745 PMC4473986

[CR103] Zhang A et al (2018) Identification of maize brace-root quantitative trait loci in a recombinant inbred line population. Euphytica 214(9):168. 10.1007/s10681-018-2203-6

[CR104] Zhong S, Xu L, Zhang Z, Li Y, Lin Z (2026) The quantitative trait locus stiff2 controls stalk bending strength and root architecture in maize. Yi Chuan Xue Bao (j Genet Genomics) 53(5):868–878. 10.1016/j.jgg.2025.11.014 41338381

[CR105] Zhu C et al (2022) Pleiotropic and nonredundant effects of an auxin importer in Setaria and maize. Plant Physiol 189(2):715–734. 10.1093/plphys/kiac115 35285930 PMC9157071

