# Supplementary material for: Genetic determinants of aerial root morphology in Sierra Mixe-derived maize
Source: Theor Appl Genet. 2026 Sep 5;139(9):260. doi: 10.1007/s00122-026-05348-w (PMC13546345; doi:10.1007/s00122-026-05348-w)
Supplement: Supplementary file 1 [file 122_2026_5348_MOESM1_ESM.docx]

**Genetic determinants of aerial root morphology in Sierra Mixe-derived maize**

Daniel Laspisa^1^*^☆^, Rubens Diogo^2☆^, Rafael Venado^2#^, Tess, Kern^2^, Natalia de Leon^3^, Jean-Michel Ané^2,3^, Jason G. Wallace^1,4^

^1^Institute of Plant Breeding Genetics and Genomics, University of Georgia, Athens, Georgia, USA

^2^Department of Bacteriology, University of Wisconsin-Madison, Madison, Wisconsin, USA

^3^Department of Plant and Agroecosystem Sciences, University of Wisconsin-Madison, Madison, Wisconsin, USA

^4^Department of Crop & Soil Sciences, University of Georgia, Athens, Georgia, USA

*Current Address: Driscoll’s of Florida, Dover, Florida, USA

^#^Current Address: International Maize and Wheat Improvement Center (CIMMYT), El Batán, State of Mexico, Mexico

^☆^These authors contributed equally to this work

Corresponding author: Jason Wallace, [jason.wallace@uga.edu](mailto:jason.wallace@uga.edu)

ORCIDs

●	DL: 0009-0000-5781-4142

●	RD: 0000-0003-2584-8158

●	REV: 0000-0002-5020-0348

●	TK: 0009-0006-2960-0829

●	NdL: 0000-0001-7867-9058

●	JMA: 0000-0002-3128-9439

●	JGW: 0000-0002-8937-6543

**APPENDIX 1**

**SUPPLEMENTARY FIGURES**

**&**

**SUPPLEMENTARY REFERENCES**

**Fig S1 MDS plots of all eight DH populations by landrace parent.** All eight double haploid populations are shown, colored by the landrace parent. The red asterisk represents PHZ51. Comparing PC2 and PC3 reveals a clear separation of the genotypes by landrace parent (PC1 represents the genetic contribution of PHZ51 and is not shown). These MDS plots show no secondary clusters of genotypes and exhibit the expected structure for three bi-parental crosses sharing a parent (PHZ51). These distributions indicate there is likely no genetic contamination in our double haploid materials.

**Fig S2 Multidimensional scaling plots separated by population.** MDS plots for our eight preliminary double-haploid populations (DH-I to DH-VIII) show no secondary clusters of genotypes and no clear subpopulation structure, indicating there is likely no genetic contamination in these doubled-haploid materials.

**Fig S3 Minor allele frequency plots separated by population.** Minor allele frequencies for all our eight preliminary double-haploid populations (DH-I to DH-VIII) are roughly normally distributed and centered on ~0.25, which is within expectations for our crossing scheme (BC_1_)

**Fig S4 Comparison of physical and genetic maps for our three populations.** Physical positions on the B73 v5 reference genome are shown on the X axis versus genetic map position on the Y axis. We do not observe any genetic markers that are far from their physical positions, indicating that our genetic map is concordant with the B73 reference genome

**Fig S5 Segregation distortion analysis across three mapping populations derived from crosses between Midwest-adapted inbreds (recurrent parents) and Southern Mexican landraces (donor lines).** For each mapping population (Pops 1, 2, and 3), whose data were arranged in 3 rectangles (A, B, and C, respectively), two graphs are shown: i) At the top of each rectangle, there is the observed recurrent parental allele frequency along the linkage map. Each point represents one marker, plotted at its genetic map position (x-axis, in cM) against the observed frequency of the recurrent parental allele across all lines in the population (y-axis, ranging from 0.5 to 1.0). Filled circles indicate markers that deviate significantly from the expected 3:1 recurrent-to-donor ratio (p < 0.05); faded circles indicate non-significant markers. Red points and red shading indicate regions where the recurrent parental allele is present at frequencies above the expectation (recurrent parental excess); blue points and blue shading indicate regions where the donor allele is more frequent than expected (donor excess). The horizontal dashed black line marks the expected recurrent parental frequency of 0.75, corresponding to the 3:1 Mendelian ratio for BC_1_-derived populations. The horizontal dotted grey line marks the 0.50 reference (equal allele frequencies). The solid trend line per chromosome represents a rolling mean of observed frequencies and is provided to aid visual identification of regions with sustained distortion. Chromosomes are displayed sequentially from Chr1 to Chr10, separated by a fixed gap of 18 cM, and labeled on the x-axis. ii) Complementarily, the lower part of each rectangle illustrates the genome-wide significance of departure from the expected 3:1 segregation ratio. Each point represents one marker plotted at its cumulative genetic map position (x-axis) against the −log₁₀-transformed p-value of the Chi-square goodness-of-fit test for departure from the expected 3:1 (recurrent : donor) ratio (y-axis). Filled circles indicate markers significant at p < 0.05; faded circles indicate non-significant markers. Alternating dark and medium grey colours distinguish adjacent chromosomes. The red dashed horizontal line marks the p = 0.05 significance threshold (−log₁₀(0.05) ≈ 1.30) and the purple dashed horizontal line marks the p = 0.001 threshold (−log₁₀(0.001) = 3.0). Consecutive runs of three or more markers exceeding the p = 0.001 threshold were defined as Segregation Distortion Regions (SDRs) and are described in the text and listed in the **Supplementary Table ST5**

**Fig S6 Diagnostic plots for the residualization procedure applied to the number of nodes forming aerial roots.** For each of the three mapping populations (rows), raw observed values (columns 1-2) and residuals after removal of the days-to-anthesis covariate (columns 3-4) are shown as overlaid histograms by environment (columns 1 and 3) and Q-Q plots (columns 2 and 4). Dashed lines in histograms indicate fitted normal curves; dashed diagonal lines in Q-Q plots indicate the theoretical normal quantile line fitted through the 25th and 75th percentiles. Shapiro-Wilk p-values for each distribution are shown in the Q-Q panel legends

**Fig S7 Diagnostic plots for the residualization procedure applied to number of aerial roots per node.** For each of the three mapping populations (rows), raw observed values (columns 1-2) and residuals after removal of the days-to-anthesis covariate (columns 3-4) are shown as overlaid histograms by environment (columns 1 and 3) and Q-Q plots (columns 2 and 4). Dashed lines in histograms indicate fitted normal curves; dashed diagonal lines in Q-Q plots indicate the theoretical normal quantile line fitted through the 25th and 75th percentiles. Shapiro-Wilk p-values for each distribution are shown in the Q-Q panel legends

**Fig S8 Diagnostic plots for the residualization procedure applied to aerial root diameter.** For each of the three mapping populations (rows), raw observed values (columns 1-2) and residuals after removal of the days-to-anthesis covariate (columns 3-4) are shown as overlaid histograms by environment (columns 1 and 3) and Q-Q plots (columns 2 and 4). Dashed lines in histograms indicate fitted normal curves; dashed diagonal lines in Q-Q plots indicate the theoretical normal quantile line fitted through the 25th and 75th percentiles. Shapiro-Wilk p-values for each distribution are shown in the Q-Q panel legends

**Fig S9 MA plots for gene expression data for 5007 maize genes located within the 37 previously mapped QTL, across tissues from maize aerial roots, internodes, and crown roots.** The raw data were compiled from publicly available maize developmental atlases (Stelpflug et al. 2016; Walley et al. 2016) hosted on the qTeller platform (<https://qteller.maizegd.org/>) (Woodhouse, Sen, et al. 2021). A) In the upper part of the image, we plotted results obtained after comparing gene expression in the aerial roots originating from the first node above ground with tissues from the fourth elongated internode up to the V9 phenological stage, which was considered the baseline (that is, the reference level). B) At the bottom, the comparison was made between aerial roots originating from the first node above ground, which were in earlier stages of elongation and thickening, and crown roots growing along the soil line, which were already fully elongated during phenological state V13 (baseline). In both plots, the Log₂ values for mean expression (x-axis) and fold change (y-axis) are shown, calculated from the available Fragments Per Kilobase of transcript per Million (FPKM) values

**Fig S10 Heat map of gene expression for 89 maize genes in tissues from aerial roots, crown roots, and internodes.** The raw data, which are publicly available on the qTeller platform (https://qteller.maizegdb.org/) (Woodhouse et al. 2021), were kindly provided by Stelpflug *et al.* (2016) and Walley *et al.* (2016) as Fragments Per Kilobase of transcript per Million (FPKM) values. They were Log_2_-transformed to facilitate data visualization. The Log_2_-transformed gene expression was plotted on a scale from blue to red, as described in the captions in the lower-right corner of the image. Differentially expressed genes (DEGs) were clustered in five groups based on their expression patterns in three tissue groups (aerial roots, crown roots, and internodes). The acronyms on the x-axis signify: V_ = maize vegetative phenological stage (represented by the letter V) followed by a number corresponding to the leaves with a visible collar (or ligule); p = pollinated plant; np = nonpollinated plant; DAP = days after pollination

**Supplementary References:**

Baldani, V.L.D., Baldani, J.I. and Döbereiner, J. (1983) “Effects of Azospirillum inoculation on root infection and nitrogen incorporation in wheat,” *Canadian journal of microbiology*, 29(8), pp. 924–929. Available at: https://doi.org/10.1139/m83-148.

Burton, A.L., Johnson, J.M., *et al.* (2014) “QTL mapping and phenotypic variation for root architectural traits in maize (Zea mays L.),” *Theoretical and applied genetics*, 127(11), pp. 2293–2311. Available at: https://doi.org/10.1007/s00122-014-2353-4.

Cai, H. *et al.* (2012) “Identification of QTLs for plant height, ear height and grain yield in maize (Zea mays L.) in response to nitrogen and phosphorus supply,” *Plant breeding*, 131(4), pp. 502–510. Available at: https://doi.org/10.1111/j.1439-0523.2012.01963.x.

Chen, G. *et al.* (2023) “Genetic basis of resistance to southern corn leaf blight in the maize multi-parent population and diversity panel,” *Plant biotechnology journal*, 21(3), pp. 506–520. Available at: https://doi.org/10.1111/pbi.13967.

Dreyfus, B. and Dommergues, Y. (1981) “Nitrogen-fixing nodules induced by Rhizobium on the stem of the tropical legume Sesbania rostrata,” *FEMS microbiology letters*, 10, pp. 313–317. Available at: https://doi.org/10.1111/J.1574-6968.1981.TB06262.X.

Gu, D. *et al.* (2017) “QTL Identification for Brace-Root Traits of Maize in Different Generations and Environments,” *Crop science*, 57(1), pp. 13–21. Available at: https://doi.org/10.2135/cropsci2016.01.0031.

Karnatam, K.S. *et al.* (2023) “Genome-Wide Meta-Analysis of QTLs Associated with Root Traits and Implications for Maize Breeding,” *International journal of molecular sciences*, 24(7), p. 6135. Available at: https://doi.org/10.3390/ijms24076135.

Ku, L.X. *et al.* (2012) “QTL mapping and epistasis analysis of brace root traits in maize,” *Molecular breeding: new strategies in plant improvement*, 30(2), pp. 697–708. Available at: https://doi.org/10.1007/s11032-011-9655-x.

Liu, Z. *et al.* (2023) “Hybrid performance evaluation and genome-wide association analysis of root system architecture in a maize association population,” *Theoretical and applied genetics*, 136(9), p. 194. Available at: https://doi.org/10.1007/s00122-023-04442-7.

Meng, L. *et al.* (2015) “QTL IciMapping: Integrated software for genetic linkage map construction and quantitative trait locus mapping in biparental populations,” *The crop journal*, 3(3), pp. 269–283. Available at: https://doi.org/10.1016/j.cj.2015.01.001.

Moussa, A.A. *et al.* (2021) “Genome-wide association screening and verification of potential genes associated with root architectural traits in maize (Zea mays L.) at multiple seedling stages,” *BMC genomics*, 22(1), p. 558. Available at: https://doi.org/10.1186/s12864-021-07874-x.

Pedrosa, F.O. and Yates, M.G. (1984) “Regulation of nitrogen fixation (nif) genes of Azospirillum brasilense by nifA and ntr (gln) type gene products,” *FEMS microbiology letters*, 23(1), pp. 95–101. Available at: https://doi.org/10.1111/j.1574-6968.1984.tb01042.x.

Pedrosa, F.O. *et al.* (2011) “Genome of Herbaspirillum seropedicae strain SmR1, a specialized diazotrophic endophyte of tropical grasses,” *PLoS genetics*, 7(5), p. e1002064. Available at: https://doi.org/10.1371/journal.pgen.1002064.

Perin, L. *et al.* (2006) “Burkholderia silvatlantica sp. nov., a diazotrophic bacterium associated with sugar cane and maize,” *International journal of systematic and evolutionary microbiology*, 56(Pt 8), pp. 1931–1937. Available at: https://doi.org/10.1099/ijs.0.64362-0.

Qiu, Y. *et al.* (1981) “Investigation of dinitrogen fixation bacteria isolated from rice rhizosphere,” *Chinese Sc bull (kexuetongbao)*, 26(26), pp. 383–384.

Robinson, A.C., Burgess, B.K. and Dean, D.R. (1986) “Activity, reconstitution, and accumulation of nitrogenase components in Azotobacter vinelandii mutant strains containing defined deletions within the nitrogenase structural gene cluster,” *Journal of bacteriology*, 166(1), pp. 180–186. Available at: https://doi.org/10.1128/jb.166.1.180-186.1986.

Setubal, J.C. *et al.* (2009) “Genome sequence of Azotobacter vinelandii, an obligate aerobe specialized to support diverse anaerobic metabolic processes,” *Journal of bacteriology*, 191(14), pp. 4534–4545. Available at: https://doi.org/10.1128/JB.00504-09.

Stelpflug SC et al. 2016. An Expanded Maize Gene Expression Atlas based on RNA Sequencing and its Use to Explore Root Development. The plant genome. 2016;9(1):lantgenome2015.04.0025. https://doi.org/10.3835/plantgenome2015.04.0025. https://doi.org/10.3835/plantgenome2015.04.0025

Sun, D. *et al.* (2022) “Genome-wide association study reveals the genetic basis of brace root angle and diameter in maize,” *Frontiers in genetics*, 13, p. 963852. Available at: https://doi.org/10.3389/fgene.2022.963852.

Sun, N. *et al.* (2020) “QTL identification in backcross population for brace-root-related traits in maize,” *Euphytica; Netherlands journal of plant breeding*, 216(2), p. 32. Available at: https://doi.org/10.1007/s10681-020-2561-8.

Venado, R.E. *et al.* (2025) “Mucilage produced by aerial roots hosts diazotrophs that provide nitrogen in Sorghum bicolor,” *PLoS biology*, 23(3), p. e3003037. Available at: https://doi.org/10.1371/journal.pbio.3003037.

Walley JW et al. 2016. Integration of omic networks in a developmental atlas of maize. Science. 2016;353(6301):814–818. https://doi.org/10.1126/science.aag1125. https://doi.org/10.1126/science.aag1125

Wang, H. *et al.* (2019) “Integrating GWAS and Gene Expression Analysis Identifies Candidate Genes for Root Morphology Traits in Maize at the Seedling Stage,” *Genes*, 10(10). Available at: https://doi.org/10.3390/genes10100773.

Woodhouse MR, Sen S, et al. 2021. qTeller: a tool for comparative multi-genomic gene expression analysis. Bioinformatics (Oxford, England). 2021;38(1):236–242. https://doi.org/10.1093/bioinformatics/btab604. https://doi.org/10.1093/bioinformatics/btab604

Woodhouse MR, Cannon EK, et al. 2021. A pan-genomic approach to genome databases using maize as a model system. BMC Plant Biology. 2021;21(1):385. https://doi.org/10.1186/s12870-021-03173-5. https://doi.org/10.1186/s12870-021-03173-5

Zhang, A. *et al.* (2018) “Identification of maize brace-root quantitative trait loci in a recombinant inbred line population,” *Euphytica; Netherlands journal of plant breeding*, 214(9), p. 168. Available at: https://doi.org/10.1007/s10681-018-2203-6.
